# 4-D Reconstruction of Fetal Left Ventricle From Echocardiography via 2.5-D Radial Segmentation and Graph-Fourier Reconstruction

**DOI:** 10.1109/TMI.2026.3707322

**Published:** 2026-08-01

**Authors:** Kamrul Hasan, Qifeng Wang, Haziq Shahard, Lucas Iijima, Nida Ruseckaite, Yihao Luo, Iris Scharnreitner, Andreas Tulzer, Bin Liu, Guang Yang, Senior Member, IEEE, Choon Hwai Yap, IEEE Member

**Affiliations:** Bioengineering Department, Imperial College London, SW7 2AZ London, U.K.; School of Software Technology, DUT, Dalian 116042, China; Bioengineering Department, Imperial College London, SW7 2AZ London, U.K.; Department of Computing, Imperial College London, SW7 2AZ London, U.K.; Bioengineering Department, Imperial College London, SW7 2AZ London, U.K.; Bioengineering Department, Imperial College London, SW7 2AZ London, U.K.; Medical Faculty and the Department of Gynaecology, Obstetrics and Gynaecological Endocrinology, Kepler University Hospital, Johannes Kepler University Linz, 4040 Linz, Austria; Medical Faculty and the Children’s Heart Center Linz, Department of Pediatric Cardiology, Kepler University Hospital, Johannes Kepler University Linz, 4040 Linz, Austria; School of Software Technology, DUT, Dalian 116042, China; Bioengineering Department and the Imperial-X, Imperial College London, W12 7SL London, U.K., also with the National Heart and Lung Institute, Imperial College London, SW7 2AZ London, U.K., also with the Cardiovascular Research Center, Royal Brompton Hospital, SW3 6NP London, U.K., and also with the School of Biomedical Engineering and Imaging Sciences, King’s College London, WC2R 2LS London, U.K.; Bioengineering Department, Imperial College London, SW7 2AZ London, U.K.

**Keywords:** 4D heart reconstruction, echocardiography, cardiac segmentation, symmetry-aware learning, graph-Fourier reconstruction

## Abstract

4D (3D over time) fetal heart reconstruction improves detection and functional assessment of congenital malformations compared with 2D methods, but remains challenging due to the lack of publicly available 4D echocardiography datasets, the burden of full 3D/4D annotations, and the computational cost of volumetric networks. To address these challenges, we introduce a 2.5D radial-slicing paradigm that converts 3D volumes into a set of angularly structured long-axis slices, inducing a consistent U-shaped anatomical appearance that facilitates manual annotation and introduces an acquisition-induced angular symmetry as an effective inductive bias. Based on this slicing, we construct FeEcho4D, the first public benchmark for 4D fetal echocardiography (52 subjects, 1845 annotated volumes). Building on this inductive bias, we propose SCOPE-Net, a symmetry-consistent, prompt-enhanced segmentation network that encodes radial symmetry via novel learnable Flip-Consistent Radial Attention and Symmetry-induced Self-distillation through inter-slice augmentation invariance, enabling label-free representation-level self-supervision. Sparse radial segmentations are subsequently reconstructed into temporally coherent 3D meshes using graph-harmonic deformation, providing a geometry-aware alternative to volumetric segmentation and voxel-based surface extraction without requiring dense 3D annotations. Extensive experiments on FeEcho4D and the public MITEA dataset show that radial segmentation outper-forms standard short-, long-axis, and volumetric views, while SCOPE-Net yields anatomically plausible 3D meshes over time. Our framework achieves an 88.8% correlation in ejection fraction, surpassing 3D volumetric segmentation (81.9%) and conventional 2D approaches (64.4%). These results indicate that geometry-aware 2.5D learning can outperform fully volumetric models for 4D fetal cardiac analysis, enabling accurate, efficient, and high-fidelity functional assessment without the need for dense 3D annotations. The FeEcho4D dataset and associated resources are publicly available at https://feecho4d.github.io/Website/

## I Introduction

**F**ETAL echocardiography is a key imaging tool for prenatal detection of Congenital Heart Disease (CHD) and assessment of cardiac function to aid clinical and parental decision-making. However, 2D fetal echocardiographic CHD screening only has a 50% average detection rate [1], and fetal echocardiography performs poorly in precision with substantial variability across studies [2], [3]. These variability issues likely stem from human error and subjectivity, which Deep Learning (DL) is well-equipped to address.

Clinically, fetal echocardiography is typically performed in 2D, as are most current DL algorithms for fetal echo [4]. However, 2D planes can miss defect locations or provide incomplete views, leading to diagnostic errors [5]. Transducer placement is also challenging, with misalignment and foreshortening causing measurement errors [6]. Moreover, 2D Ejection Fraction (EF) estimation assumes circular cross-sections and uses Simpson’s rule, which can introduce errors in cases with asymmetric wall thickness or cardiac malformations. In contrast, 3D echo captures the full heart volume without requiring precise alignment and can improve detection and functional assessment. Yet, although 3D/4D fetal echo is available via Spatio-temporal Image Correlation (STIC) acquisitions, it remains underused due to challenges in visualization and quantification. DL-based 3D/4D cardiac reconstruction offers a promising solution to these limitations.

**Fig. 1:**
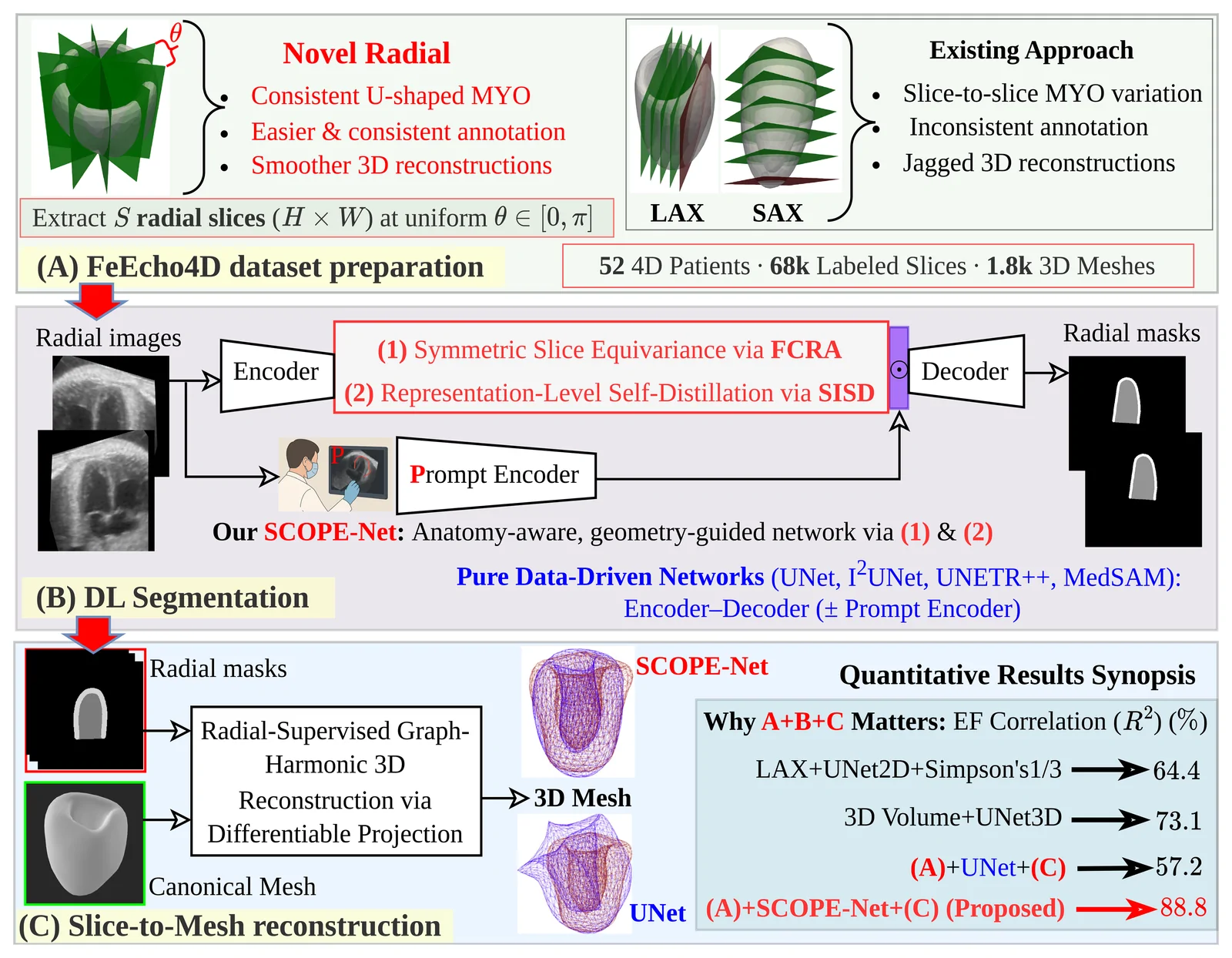
Proposed full-stack framework from data preparation to mesh reconstruction. (A) Radial FeEcho4D data preparation (Section III-A). (B) Segmentation network incorporating geometric priors via Flip-Consistent Radial Attention (FCRA) (Algorithm 1) and augmentation-invariance self-distillation via Self-induced Self-distillation (SISD) (Section III-B). (C) Sparse radial slice-to-3D reconstruction (Section III-C). A purely data-driven UNet produces anatomically implausible meshes (ground truth: red wireframe and estimated: blue wireframe), leading to poor EF estimation (Table V).

For reconstructing fetal cardiac geometry from 3D/4D images, non-DL methods [7] are time-consuming. To date, no DL-based framework has been developed due to (1) the lack of a public 3D/4D dataset and (2) the inherent difficulty of 3D/4D segmentation. Fetal images often suffer from poor quality due to anisotropic resolution, acoustic shadowing, and signal loss, complicating image processing. Moreover, preparing 3D ground-truth masks is both time-consuming and challenging, as it requires ensuring accuracy and inter-slice consistency. A specific challenge arises during slice-by-slice labeling on longitudinal views, as it is difficult to decide when to transition from a U-shaped Myocardial (MYO) structure to a circular blob near the lateral ends (Fig. 1A). Additionally, 3D networks are computationally expensive and challenging to train. To address the first factor, we introduce FeEcho4D, a benchmark 4D fetal echo dataset. To address the second, we present a 3D Left Ventricular (LV) reconstruction over time framework via 2.5D radial segmentation, based on radial slicing instead of Cartesian image slicing (Fig. 1A), and novel symmetry-aware learning via geometric inductive bias (Fig. 1B).

Radial slicing (Fig. 1A) is a promising yet underexplored alternative to traditional Cartesian slicing, extracting 2D slices by rotating the slicing plane around the LV’s longitudinal axis at fixed angular intervals, *θ* ∈ [0, *π*]. This cylindrical-coordinate acquisition provides structured coverage around the heart, enabling consistent LV visualization from multiple orientations. Interestingly, radial slicing introduces angular symmetry: slices at angles *θ* and *π* − *θ* exhibit approximately symmetric anatomy (see Fig. 3). This property has two key implications: (1) it introduces a novel built-in *geometric inductive bias* (Section III-B1) that can be explicitly modeled in the network (Fig. 1B) as a *spatial anatomical prior*, unlike purely data-driven DL models lacking anatomical priors [8], [9], [10], [11], [12], [13], [14]; and (2) it yields consistent, interpretable U-shaped MYO contours (see example slices in Fig. 3), which are easy to annotate even under noise or signal dropout.

To exploit this novel geometric inductive bias, we propose a novel spatial prompt-driven segmentation framework with novel Flip-Consistent Radial Attention (FCRA) for equivariant feature aggregation across symmetric pairs. To enforce representation-level consistency, we introduce a novel Self-induced Self-distillation (SISD) mechanism via our Inter-Slice Augmentation Invariance (ISAI) learning. Our ISAI leverages anatomical symmetry to define semantically grounded positive pairs based on domain-specific inductive bias, unlike other Self-supervised Learning (SSL) methods. Our SISD is fully self-contained, requiring no target network or added memory. SISD’s supervisory signal emerges from the inter-play between architectural symmetry and acquisition-aware augmentations. Our formulation and design in Fig. 1 (B) thus maximize agreement across anatomically symmetric, independently augmented slices, yielding symmetry-aware, augmentation-invariant representations.

Finally, we propose to use the Graph-Harmonic Deformation (GHD) [18] mesh morphing approach to model heart shape, enabling high-quality 3D reconstruction even from sparse or imperfect 2D segmentations. We propose that our full-stack framework is more effective than segmenting dense Cartesian slices followed by other reconstruction algorithms: Marching Cubes [19], non-rigid Coherent Point Drift (NrCPD) [20], and non-rigid Iterative Closest Point (NrICP) [21]. Key aspects of the contributions are summarized in Table I.

## II Related Work

Currently, public fetal cardiac datasets are limited to 2D frames with limited spatial or temporal context. Stoean et al. [22] released 1,040 labeled frames. Liang et al. [23] introduced 1,083 images with 15 anatomical labels. EchoNet-Peds [24] offers 7,643 cine clips from 4,467 pediatric studies, with expert LV contours and EF values. To address the lack of a 3D/4D fetal echo dataset, we introduce FeEcho4D, which includes 1,845 annotated 3D volumes (68,265 radial slices).

Since UNet [8], numerous extensions have improved contextual modeling, including dilations [25], residual paths [26], and automated pipelines (nnUNet) [9]. Segment Anything (SAM) [13] is a pioneering interactive segmentation model, enabling region-focusing through diverse user prompts. Transformer-based models have recently emerged, combining global attention with CNN locality, for example, nnFormer [27]. In echocardiography, Zhao et al. [12] introduced BATNet, a semi-supervised transformer to enforce boundary consistency. Xue et al. [28] incorporated motion information via optical flow and introduced an orientation-congruency constraint to ensure spatiotemporal alignment. Wei et al. [29] proposed MCLAS for multi-task learning with view classification and EF prediction. Hasan et al. [30] introduced a temporal attention, where each frame’s segmentation benefits from context across the sequence, improving temporal coherence. Shaker et al. [10] presented UNETR++, a 3D transformer model that employs a paired attention block with spatial and channel attention branches to enhance discriminative feature learning. Existing segmentation models are data-driven, relying on Cartesian short- and long-axis (SAX, LAX) views or 3D volumes, lacking explicit anatomical priors, and often struggle in ambiguous or low-quality regions. We address this by introducing a novel radial slicing approach and a geometry-adaptive framework that models angular symmetry as a *spatial anatomical prior*. Radial slices also yield consistent U-shaped MYO contours (see example slices in Fig. 3) that are easier to annotate or predict, even with poor images.

Recent work has shown that attention mechanisms can enhance focus on relevant anatomical structures. Spatial and channel attention [31] prioritize informative features and locations. Temporal attention has also been explored across domains; in natural video, Lu et al. [32] employed co-attention to maintain object consistency across frames and later extended it to 3D echocardiographic LV segmentation [33]. More recently, Hasan et al. [34] proposed a feedback attention mechanism that generates co-attention maps of residual spatial errors to improve self-supervision. In contrast, our attention module, FCRA, enforces alignment between symmetric radial slices across augmentations, capturing angular symmetry from the acquisition geometry for a consistent feature representation.

In 3D mesh reconstruction from voxelated labels, the traditional Marching Cubes algorithms [19] generate staircase artifacts, which require excessive smoothing that reduces image fidelity. The correspondence-based non-rigid registrations using NrCPD [20] and NrICP [21] are sensitive to sparse or noisy segmentation, non-uniform point density, and correspondence ambiguity, leading to unstable deformations of the fixed template. To improve this, we previously proposed the GHD shape model [18], which employs Laplacian eigenmodes to ensure mesh smoothness without explicit regularization, and a DVS method [35], which enables gradient-based mesh optimization from 2D supervision. Together, DVS and GHD form a fully differentiable framework for 3D mesh reconstruction. Unlike registration-based morphing approaches [20], [21], [36], GHD+DVS does not rely on large annotated datasets and remains robust under sparse observations. We adopt this approach in our pipeline (Fig. 1C) for 3D mesh reconstruction from annotated (and DL segmented) 2.5D radial slices. Table I presents the summary of our contributions along four key aspects compared to representative prior work.

## III Methodology and Materials

### A Data Preparation: Slicing, Annotation, Reconstruction

#### 1) Radial Slice Extraction

Given a 4D sequence **V** ∈ ${\mathbb{R}}^{T\times H^{\prime }\times W^{\prime }\times D^{\prime }},$, where *H*′ × *W*′ × *D*′ denotes the spatial resolution, we extract a set of radial slices at each time point *t* ∈ ℝ {1, …, *T*}. Specifically, we uniformly sample the angular domain *θ* ∈ [0, *π*] at 5° intervals, yielding *S* = 37 radial slices per volume, where each slice intersects the selected LV center (*C_x_, C_y_*). Here, *C_x_* and *C_y_* denote the lateral and apical-basal in-plane coordinates of the LV center, defined on the selected slice (*Z_m_*) of the slice-stack (through-plane) axis (*D*′). The LV center (*C_x_, C_y_*) and selected slice (*Z_m_*) are manually defined in the first frame and then fixed in space in all subsequent frames. In practice, this initialization is stable, as cardiac motion remains largely centered on the LV cavity, resulting in limited inter-frame displacement. This is supported by the sensitivity analysis (Section V-B2), which shows that such deviations have a limited impact, indicating robustness even under poor LV rotational axis specification.

Algorithm 1 **Pytorch-Like FCRA Module’S Steps**

**Input:** Feature tensor *f* ∈ R^*B*×*S*×*C*×*h*×*w*^

**Output:** FCRA-enhanced tensor $\hat{f}\in {\mathbb{R}}^{B\times S\times C\times h\times w}$

1. **Reshape input across spatial locations:**
  $\tilde{f}\leftarrow f$. permute(0, 3, 4, 1, 2).reshape(−1, *S, C*)
2. **Linear projections to obtain query, key, and value:**
  $$Q=\tilde{f}W_{q},\;K=\tilde{f}W_{k},\;V=\tilde{f}W_{v}$$
3. **Flip keys and values to incorporate inductive bias symmetry, stated as:**
  $$f_{\theta_{s}}(i,j)\approx f_{\pi -\theta_{s}}(i,w-1-j)K_{\text{flip}}\leftarrow \text{Flip}(K),V_{\text{flip}}\leftarrow \text{Flip}(V)$$
4. **Compute attention weights (scaled dot-product):**
  $$A=\text{softmax}\left(\frac{QK_{\text{fip}\;}^{\text{T}}}{\sqrt{C}}\right)$$
5. **Apply residual connection on attended features:**
  $${\hat{f}}_{\text{flat}}=A\cdot V_{\text{flip}}+\tilde{f}$$
6. **Reshape back to original shape:** $\hat{f}={\hat{f}}_{\text{flat}}$. view(*B, h, w, S, C*).permute(0, 3, 4, 1, 2)
7. **return** $\hat{f}\in {\mathbb{R}}^{B\times S\times C\times h\times w}$

Given anisotropic voxel spacing [*s_x_, s_y_, s_z_*], we construct a regular 3D meshgrid of spatial coordinates **X** = {(*x_i_, y*_*j*_, *z_k_*)}, where *x_i_* = *i* · *s_x_, y*_*j*_ = *j* · *s_y_, z_k_* = *k* · *s_z_*, and *i, j, k* ∈ [1, *W*′], [1, *H*′], [1, *D*′]. This grid, **X**, is translated such that the rotation center aligns with the point [*C_x_* · *s_x_, C_y_* · *s_y_, Z_m_* · *s_z_*], yielding a centered coordinate set, **X**^*C*^. For each angular direction *θ*, we apply a 3D rotation about the *y*-axis, and the transformed coordinates are then mapped back to the original reference frame, as in (1).

(1) $$\begin{array}{c} X^{\text{target}\;}=R_{y}(\theta )X^{𝒞}+\left[C_{x}s_{x},C_{y}s_{y},Z_{m}s_{z}\right],\\ R_{y}(\theta )=\left[\begin{array}{ccc} \cos\theta & 0 & \sin\theta \\ 0 & 1 & 0\\ -\sin\theta & 0 & \cos\theta \end{array}\right],\\ \theta =\frac{\pi s}{S-1},\;s\in [0,S-1]. \end{array}\}$$

The angular parameter *θ* is used solely to rotate the sampling plane around the fixed LV long axis, defining a cylindrical coordinate system, and to index the resulting slices. The initial angular reference (*s* = 0) can be defined as the radial slice that maximizes the image symmetry between *θ* and *π* − *θ*, which can be determined by a correlation map between all combinations of radial slices (see Fig. 3 and Section III-B1).

Finally, cubic interpolation is applied to each volume **V**^*t*^, for *t* ∈ {1, …, *T*}, at the transformed coordinates, yielding a rotated volume $V_{\theta }^{t}$. From each rotated volume, we extract the axial slice at *Z_m_*, giving a 2D radial slice $I_{\theta_{s}}^{t}$. The collection of slices across all sampled angles forms a radial stack $V_{\theta }^{t}\in {\mathbb{R}}^{S\times H\times W}$ for each time point *t*, which extends across time to produce the full 4D radial representation **V**_*θ*_ ∈ ℝ ^*T*×*S*×*H*×*W*^ of the given $V\in {\mathbb{R}}^{T\times H^{\prime }\times W^{\prime }\times D^{\prime }}$. The sensitivity of the geometric parameters *C_x_, C_y_*, and *Z_m_* used to construct **V**_*θ*_ is evaluated in Section V-B2.

#### 3) Manual Annotation

To obtain high-quality masks, we developed an annotation tool that supports volume loading, manual delineation, real-time precision checking via mask overlay, temporal navigation to account for cardiac motion (particularly useful for cases with signal dropout), and annotation toggling for refinement (demos on project website). Annotators manually segmented all *S* radial slices at ED and ES, and underwent iterative refinement and cross-validation to the satisfaction of a pediatric cardiologist, yielding two 3D masks for each case, $M_{\theta }^{t}\in {\mathbb{R}}^{S\times H\times W},$ ∀*t* ∈ {ED,ES}.

#### 3) Motion Tracking

To obtain segmentations for all time points, *M*_*θ*_ ∈ ℝ ^*T*×*S*×*H*×*W*^, we adopted our previous spatial B-spline of temporal Fourier (BSF) motion tracking frame-work [37]. Given a radial cine sequence **V**_*θ*_ and sparse ES/ED masks, $M_{\theta }^{t}\in {\mathbb{R}}^{S\times H\times W},$ we estimate pairwise **d**_*t*→*t*+1_ and anchor-based **d**_0→*t*+1_ deformation fields. A reference grid **X**_0_ is initialized from $M_{\theta }^{ED},$ and propagated using a convex combination of local and anchor displacements, as in (2).

(2) $$X_{t+1}=w(t)\left(X_{t}+d_{t\to t+1}\right)+(1-w(t))\left(X_{0}+d_{0\to t+1}\right),$$

where *w*(*t*) ∈ [0, 1] is a time-dependent weight balancing local continuity and global anchor stability. The temporal Fourier formulation ensured cyclicity of motion, while B-splines ensured spatial consistency. Control coefficients are optimized following [37].

#### 4) Further Refinement

The dense binary mask *M*_*θ*_ is refined by multiple annotators using our annotation tool, with pediatric cardiologist approval to ensure spatial, temporal, and interrater consistency. To further enforce anatomical regularity, we apply a skeleton-based post-processing step. For each slice, the largest connected component is skeletonized and extrapolated, and the mean half-width is estimated as in (3).

(3) $$h_{s}=└\frac{1}{\left|S_{s}\right|}\underset{p\in S_{s}}{\sum }\underset{q\in \bar{M_{s}^{\prime }}}{\min}\|p-q{\|}_{2}+\frac{1}{2}┘,$$

where *S_s_* is the skeleton point set and $\bar{M_{s}^{\prime }}$ the mask complement. The estimated *h_s_* defines the local MYO half-thickness. A uniform mask is then reconstructed via distance transform thresholding and optional morphological smoothing, which regularizes wall thickness and suppresses artifacts. The corrected masks *M*_*θ*_ ∈ ℝ ^*T*×*S* ×*H*×*W*^ are finally used for 3D mesh reconstruction (Section III-C), yielding a temporally coherent sequence of *T* ground-truth meshes. Further details are available at https://feecho4d.github.io/Website/

### B Proposed Segmentation Network

We propose **SCOPE-Net**, a novel segmentation frame-work designed for radial imaging. SCOPE-Net incorporates *bilateral attention* to model anatomical symmetry across angularly mirrored slice pairs (*θ, π* − *θ*) and couples *spatial prompts* for minimal but effective supervision [13]. To enforce representation-level consistency, we introduce the SISD framework with a novel ISAI objective that aligns feature representations between independently augmented, symmetrically paired slices.

**Fig. 2:**
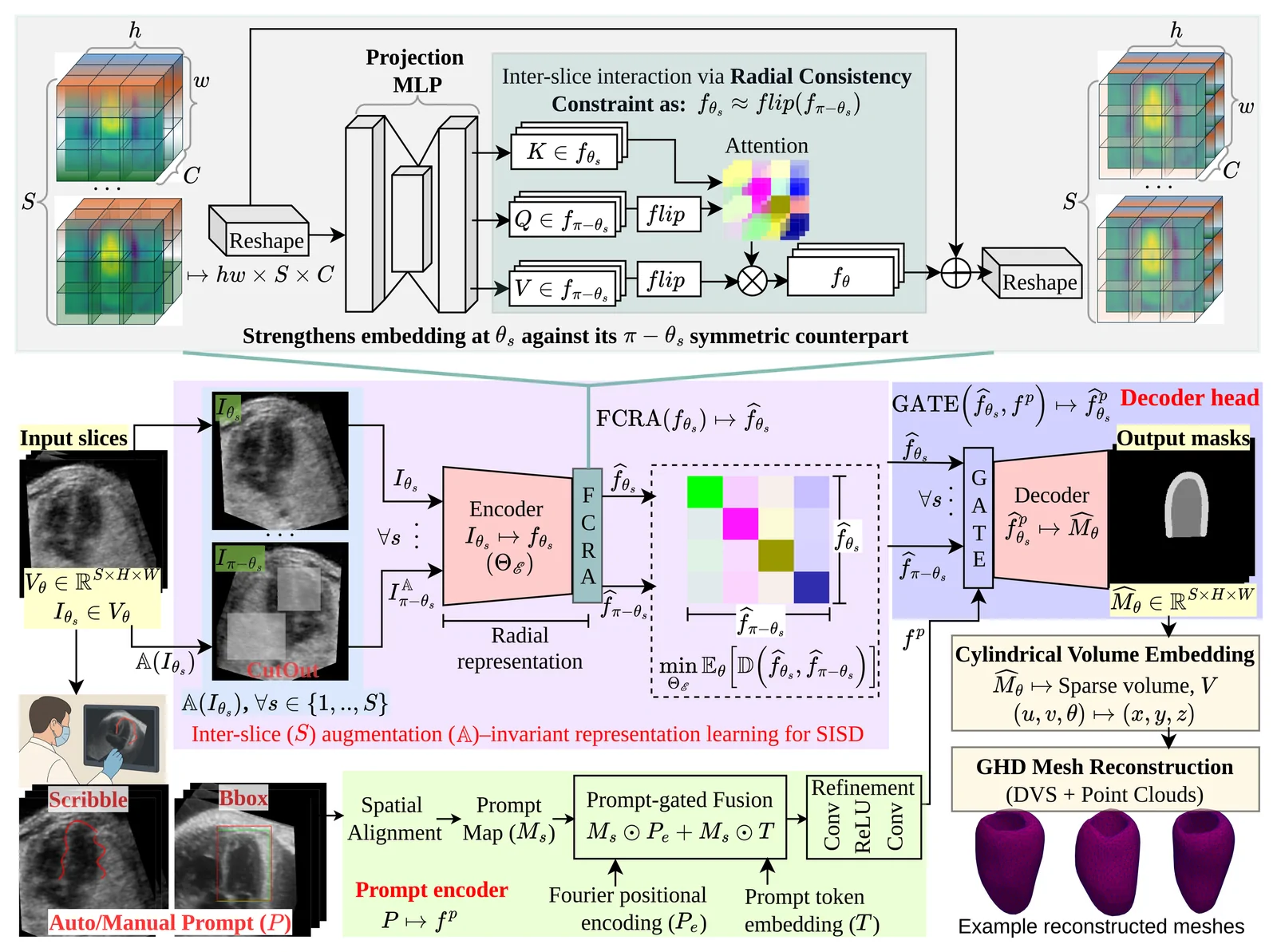
Overview of SCOPE-Net, a symmetry-aware segmentation framework that exploits acquisition-induced radial geometry. Angularly symmetric slices are processed with FCRA to enforce flip-consistent attention, while prompt-based conditioning injects spatial priors for ambiguous regions. SISD, guided by an ISAI objective, leverages independently augmented symmetry pairs for representation-level self-distillation, improving robustness and alignment. The resulting radial segmentations are embedded into a cylindrical volume representation and further processed with GHD-based mesh reconstruction to produce anatomically consistent 3D cardiac meshes from sparse radial views.

Fig. 2 shows our proposed SCOPE-Net, where ISAI improves generalization in low-supervision settings via leveraging *Radial Inductive Bias* (III-B1). Given a radial slice $I_{\theta_{s}}\in {\mathbb{R}}^{H\times W},$∀_*s*_∈{1,&*S*} the network predicts a mask ${\hat{M}}_{\theta_{s}}=ℱ\left(I_{\theta_{s}};\text{Θ}\right),$ where *ℱ* is a learnable function parameterized by Θ. The network adopts a UNet-style architecture with skip connections [8], where the encoder (*E*) extracts multiscale features and the decoder (*D*) reconstructs the segmentation mask. At each resolution level *l* ∈ *L*, encoder features are computed recursively as: *f* ^*l*^ = *E*^*l*^(*f* ^*l*−1^), with $f^{0}=I_{\theta_{s}},$ and the decoder predicts ${\hat{Μ}}_{\theta_{s}}=𝒟\left(f^{L}\right)$. To enhance feature propagation and training stability, we replace standard con-volutional blocks with residual blocks. Each residual block contains two 3 × 3 convolutions with batch normalization and ReLU activation, along with an identity or 1 × 1 projection shortcut to match dimensions. This design improves gradient flow, facilitates deeper network optimization, and preserves fine-grained spatial information across scales.

#### 1) Radial Inductive Bias

**Fig. 3:**
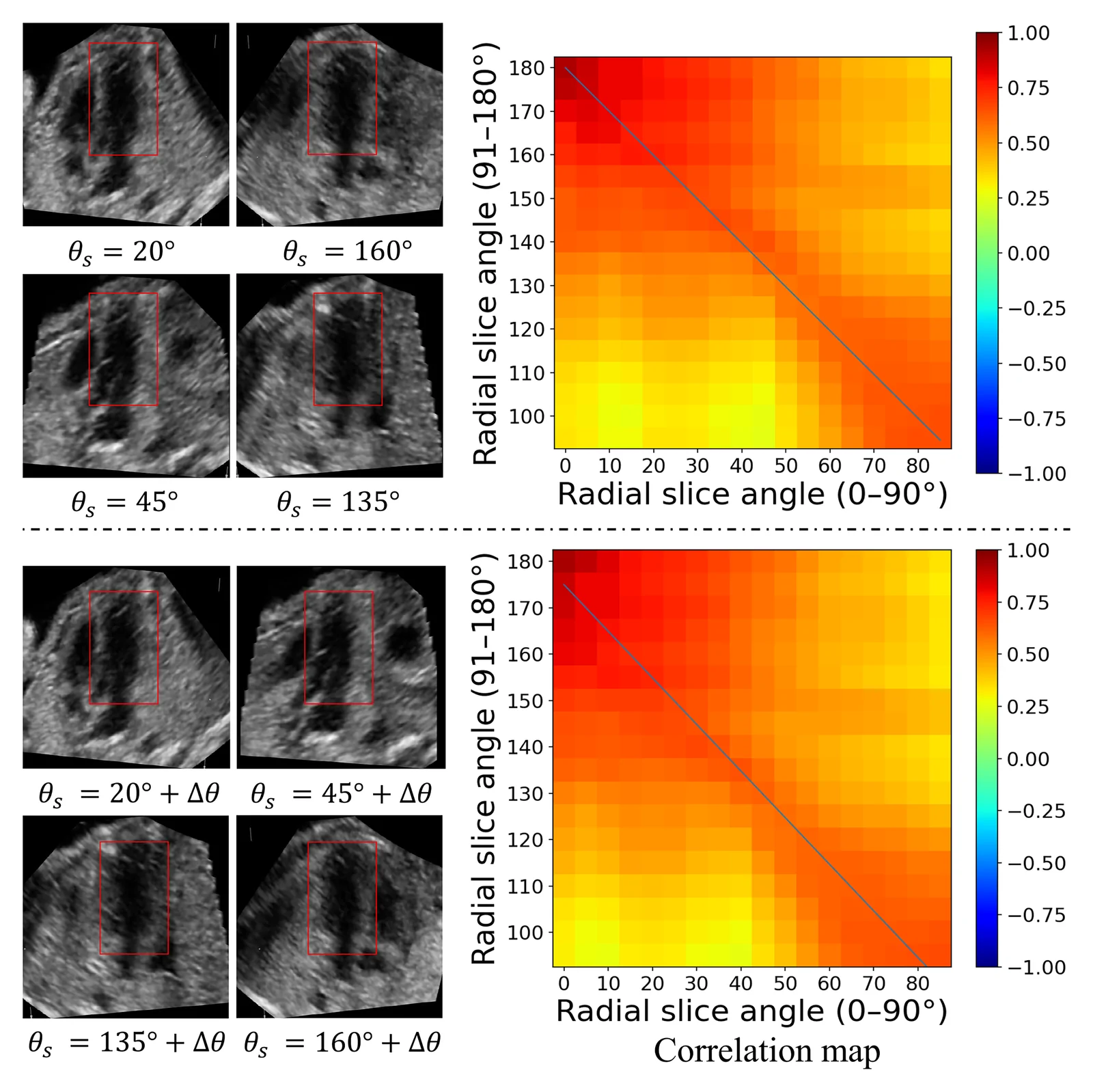
**Top two rows:** Angularly paired radial slices (consistent U-shaped myocardial appearance) at angles θ and π − *θ* qualitatively demonstrate mirror symmetry. The correlation map (right) exhibits a clear diagonal ridge, indicating high consistency between mirror-paired slices. **Bottom two rows:** The same analysis but with a shifted initial angular reference (Δ*θ* = 5°). The diagonal ridge in the correlation map remains clearly visible, and the plane of symmetry remains extractable. The blue diagonal line represents a regression line of the maximum correlations.

Recalling Section III-A1, each slice $I_{\theta_{s}}\in {\mathbb{R}}^{H\times W},$ acquired at angle *θ_s_*, is spatially indexed by pixel coordinates *i* ∈ [0, *H*−1] and *j* ∈ [0, *W*−1]. Due to the circular acquisition geometry and the approximate bilateral symmetry of cardiac anatomy in long-axis views, the anatomical structure observed at angle *θ_s_* approximately corresponds to a horizontally mirrored version of the structure seen at angle *π* − *θ_s_*. In particular, paired slices often depict anatomically symmetric regions, for example, the lateral wall at *θ_s_* and the septal wall at *π* − *θ_s_*, mirrored across the vertical axis, as shown in Fig. 3. We formalize this *Radial Inductive Bias* as in (4).

(4) $$I_{\theta_{s}}\left(i,j\right)\approx I_{\pi -\theta_{s}}\left(i,W-1-j\right).$$

The validity of the approximation in (4) is empirically supported by the correlation analysis (between all combinations of radial slices) in Fig. 3, which exhibits a strong diagonal structure indicating the existence of the radial symmetry between *θ* and *π* − *θ*. Fig. 3 (bottom rows) further demonstrates that the diagonal structure can easily be extracted from simple regression (blue line), even when the plane of symmetry does not align with the imaging plane.

To respect this domain-specific geometric prior, as stated in (4), we enforce that the encoder with parameters of Θ_*ε*_ produces consistent features for symmetric slices, up to a horizontal flip. Specifically, for the encoder, we define *Consistency Constraint*, as in (5).

(5) $$\begin{array}{c} {ℰ}_{{\text{Θ}}_{ℰ}}\left(I_{\theta_{s}}\right)\left(i,j\right)\approx {ℰ}_{{\text{Θ}}_{ℰ}}\left(I_{\pi -\theta_{s}}\right)\left(i,w-1-j\right),\;w=W/16\\ \Leftrightarrow {ℰ}_{{\text{Θ}}_{ℰ}}\left(I_{\theta_{s}}\right)\approx {\text{flip}}_{W}\left({ℰ}_{{\text{Θ}}_{ℰ}}\left(I_{\pi -\theta_{s}}\right)\right), \end{array}$$

where flip_*W*_ (·) denotes horizontal flipping along the width axis. This yields an ***equivariance constraint***: the model’s predictions should remain consistent (modulo spatial reversal) when given symmetrically opposed views. We incorporate this constraint (1) into the architecture through the bilateral FCRA module (III-B2) and (2) into the learning objective ISAI in SISD (III-B3). Together, these components in our SCOPE-Net embed angular-spatial symmetry as a strong inductive bias, enabling data-efficient and anatomically plausible segmentation.

#### 2) Flip-Consistent Radial Attention

Our novel FCRA module (Algorithm 1) encodes domain-specific inductive biases by explicitly modeling geometry priors within the attention computation. Given feature tensor *f* ∈ ℝ ^*B*×*S* ×*C*×*h*×*w*^ is reshaped to $\tilde{f}\in {\mathbb{R}}^{Bhw\times S\times C}$ to compute the attention across the angular *S* -dimension independently at each spatial location. To incorporate *angular and spatial symmetry*, the key and value sequences are flipped before attention is applied. Scaled dot-product attention is then computed across the angular sequence, and the attended features are combined with the original features via a residual connection. This formulation embeds symmetry-aware equivariance directly into the architecture, enabling robust learning in anatomically structured domains. Unlike standard attention, FCRA explicitly models such geometric symmetries, making it particularly suitable for radial echocardiography or other radial imaging modalities, where symmetric slice pairs are expected by design.

#### 3) Symmetry-Induced Self-Distillation

Our formulation yields an emergent self-supervised learning framework for representation-level consistency, called *SISD*. This behavior emerges from the interaction between the architectural inductive bias and stochastic slice-wise augmentations. The augmentations 𝔸(·), including random masking, horizontal flipping, rotation, multiplicative noise, Gaussian blurring, random-sized cropping, motion blur, and random brightness adjustment, are applied independently to each angular slice. Our SISD setup forces the model to resolve semantic consistency between geometrically aligned but visually perturbed views, resembling self-distillation. As a result, the encoder is incentivized to learn augmentation-invariant, inter-slice symmetry-aware representations, referred to as ISAI. Unlike conventional SSL frameworks [16], our novel ISAI in the SISD emerges organically from the model’s structural bias and the domain-specific pairing of angularly opposed slices.

To achieve this with ISAI, we enforce consistency between the feature maps of paired slices, effectively minimizing feature discrepancy across symmetric angular views. This alignment implicitly treats the pair$\left(ℰ\left(𝔸(I_{\theta_{s}}\right)\right),$ flip_*W*_ $\left(ℰ\left(𝔸\left(I_{\pi -\theta_{s}}\right)\right)\right)$as a *positive pair*, analogous to positive pairs in contrastive learning [16], yet generated deterministically via acquisition geometry. We introduce the *Radial Flip-Equivariance (RFE*) constraint for such minimization, which penalizes discrepancies 𝔻(·) (here, cosine similarity in (8)) between angularly opposed slices, expressed as an expectation 𝔼 over slice angles, as in (6).

(6) $$\underset{{\text{Θ}}_{ℰ}}{\min}{𝔼}_{\theta_{s}\in [0,\pi ]}\left[𝔻\left(ℰ\left(𝔸\left(I_{\theta_{s}}\right)\right),flip_{W}\left(ℰ\left(𝔸\left(I_{\pi -\theta_{s}}\right)\right)\right)\right)\right].$$

By applying this constraint at the encoder level, we embed anatomical reflectional symmetry directly into the learned representations. This encourages stronger self-supervised feature alignment across angular views and introduces a robust inductive bias in low-supervision settings.

Moreover, in the ISAI setting within the SISD, under slice-wise augmentation, the FCRA module computes angular attention between $ℰ\left(𝔸\left(I_{\theta_{s}}\right)\right)$ and the horizontally flipped features of $ℰ\left(𝔸\left(I_{\pi -\theta_{s}}\right)\right).$. As detailed in Algorithm 1, the *Q, K*, and *V* tensors are computed from these augmented feature maps, with spatial mirroring applied to the key and value to align the anatomical content. This attention mechanism enforces equivariant feature aggregation across symmetric slices, constraining the model to extract structurally consistent representations despite augmentation-induced perturbations.

#### 4) Interactive Conditioning via Spatial Prompts

In addition to the bilateral FCRA module, SCOPE-Net incorporates spatial prompt-guided conditioning to modulate encoder features using bounding-box or scribble prompts, encoding both spatial location and semantic context to guide targeted refinement in challenging regions [13], [14]. Each prompt *P*(*H_f_, W_f_*) is first converted into a binary mask **M**_*s*_ to localize the region of interest. To enrich spatial priors beyond binary localization, we apply a random Fourier positional encoding to normalized spatial coordinates **U**(*i, j*) = [*j/W_f_, i/H_f_*], projecting them into a high-dimensional sinusoidal space, as in (7).

(7) $$\begin{array}{c} Z(i,j)=2\pi U(i,j)G,\\ P_{e}(i,j)=[\sin Z(i,j),\cos Z(i,j)], \end{array}$$

where **G** ∈ ℝ ^2×*D*^ is a fixed Gaussian projection matrix. The resulting encoding $P_{e}\in {\mathbb{R}}^{H_{f}\times W_{f}\times 2D}$ captures smooth positional variations and introduces global spatial awareness, analogous to positional encodings used in vision transformers [38]. Each bounding-box prompt is further associated with a learnable semantic embedding **T** ∈ ℝ^*C*^, which is broadcast spatially and fused with the masked positional encoding to form a dense prompt representation: **f** = **P**_*e*_ ⊙ **M**_*s*_ + **T** ⊙ **M**_*s*_, where ⊙ denotes elementwise multiplication. To enforce local smoothness and mitigate box-edge discontinuities, the prompt representation is refined using a shared two-layer convolutional block: **f** ^*p*^ = Conv_3×3_ ReLU Conv_3×3_(**f**), yielding a dense prompt feature map **f**^*p*^ ∈ ℝ ^*B*×*C*×*h*×*w*^, where *h, w* = *H_f_* /16, *W_f_* /16. This refinement acts as a spatial low-pass filter, promoting continuity across prompt boundaries and suppressing high-frequency artifacts.

To embed prompts in the feature stream, we apply an element-wise multiplicative gating operation (⊙), as $\hat{ℰ}\left(I_{\theta_{s}}\right)=ℰ\left(I_{\theta_{s}}\right)⊙{𝒫}_{e}(p).$. The modulated representation $\hat{ℰ}\left(I_{\theta_{s}}\right)$ is then forwarded to the decoder head. This mechanism enables the prompt to function as a soft spatial gate, enhancing activations in the user-specified region while suppressing irrelevant background. Instead of relying solely on architectural priors, our design allows dynamic adaptation through user interaction, providing robustness even in the presence of structural ambiguity, image degradation, and sudden signal dropout.

#### 5) Computational Overheads

Given a radial volume **V**_*θ*_ ∈ ℝ ^*B*×*S* ×*C*×*H*×*W*^, the 2D UNet processes all slices via batch broadcasting, reshaped to $V_{\theta }^{\prime }\in {\mathbb{R}}^{(B\cdot S)\times C\times H\times W},$ yielding FLOPs scaling as 𝒪(*Lk*^2^*C*^2^*S HW*), where *L* is the number of layers and *k* the kernel size. FCRA is applied at the bottleneck to introduce cross-slice interactions for self-distillation with only 𝒪(3*C*^2^) parameters and 𝒪(*S* ^2^*Chw*) FLOPs, where *h, w* « *H, W*. In contrast, a 3D UNet with volumetric kernels yields 𝒪(*Lk*^3^*C*^2^*S HW*) complexity. Thus, the 3D UNet remains significantly more expensive, while our design achieves near-2D efficiency while capturing 3D-like (or even better) spatial structure across slices.

#### 6) Loss Function

Our RFE loss is applied at the bottleneck feature level to explicitly enforce anatomical symmetry in the learned feature representations and to maintain consistency between structurally symmetric yet independently augmented views. The RFE loss imposes equivariance through geometric pairing rather than implicit encoding. Let *S* denote the total number of radial slices. The loss is formulated in (8).

(8) $${ℒ}_{\text{rfe}}=1-\frac{1}{S}\overset{S}{\underset{s=1}{\sum }}\frac{ℰ{\left(I_{\theta_{s}}\right)}^{⊤}{\text{flip}}_{W}\left(ℰ\left(I_{\pi -\theta_{s}}\right)\right)}{∥ℰ\left(I_{\theta_{s}}\right)∥\cdot ∥{\text{flip}}_{W}\left(ℰ\left(I_{\pi -\theta_{s}}\right)\right)∥}.$$

We also use supervised spatial losses, such as Dice score and cross-entropy, to exploit their complementary properties [10]. The final loss (ℒ) is a weighted sum of these terms, as defined in (9).

(9) $$\begin{array}{c} ℒ=\lambda_{\text{dice}}\cdot {ℒ}_{\text{dice}}+\lambda_{\text{ce}}\cdot {ℒ}_{\text{ce}}+\lambda_{\text{rfe}}\cdot {ℒ}_{\text{rfe}},\;\;\text{where}\;\\ {ℒ}_{\text{dice}}=1-\underset{i\in 𝒞}{\sum }\frac{2\left|M^{i}\cap {\hat{M}}^{i}\right|+\epsilon }{\left|M^{i}\right|+\left|{\hat{M}}^{i}\right|+\epsilon }\\ {ℒ}_{\text{ce}}=-\frac{1}{P}\underset{x\in P}{\sum }\left(M^{x}\log{\hat{M}}^{x}+\left(1-M^{x}\right)\log1-{\hat{M}}^{x}\right), \end{array}$$

where 𝒞 denotes the number of segmentation classes, *ϵ* provides numerical stability to prevent division by zero, and *P* is the number of pixels in true (*M*) and predicted $\left(\hat{\text{M}}\right)$ masks. We selected the loss weights {*λ*_dice_, *λ*_ce_, *λ*_rfe_}via random search using a held-out validation split from the training data. Specifically, we sampled *λ*_dice_, *λ*_ce_ ∈ [0.5, 2.0] and *λ*_rfe_ ∈ [0.01, 0.5] over *N* trials, training each configuration under an identical optimization protocol. The final weights were selected based on the configuration that maximized the mean validation DSC. This procedure resulted in *λ*_dice_ = 1.0, *λ*_ce_ = 1.0, and *λ*_rfe_ = 0.1.

### C Graph-Harmonic 3D Mesh Reconstruction

Prior to mesh reconstruction, we perform *Cylindrical Volume Embedding* to lift the radial slice representation into a common 3D coordinate system and enforce geometric consistency across views. Given binary masks $M_{\theta }^{t}\in {\mathbb{R}}^{S\times H\times W}$ for each time *t* ∈ *T* sampled at discrete angles *θ_s_* ∈ [0, *π*], each slice is embedded into a Cartesian volume via a cylindrical coordinate mapping. As illustrated in Fig. 2, each radial slice is rotated around the LV center and projected into a shared 3D space according to its acquisition angle. Specifically, for each pixel $(u,v)\in M_{\theta_{s}}^{t},$ its spatial location is given by (10):

(10) $$x=x_{0}+u\cos\theta_{s},\;y=y_{0}+u\sin\theta_{s},\;z=v+z_{0},$$

where (*x*_0_, *y*_0_, *z*_0_) denotes the LV-centered reference frame. This results in a sparse cylindrical volume *V*^*t*^ that encodes acquisition geometry across angular views, from which 3D point sets are subsequently extracted to guide mesh reconstruction.

From this geometrically consistent representation, we extract MYO and background point clouds $P_{\text{MYO}}^{t},P_{\text{BG}}^{t}\in {\mathbb{R}}^{N\times 3}$ to constrain mesh reconstruction. A canonical template mesh ℳ_0_ ∈ ℝ ^*V*×3^ is deformed using learnable spectral coefficients *φ*^*t*^ ∈ ℝ ^*B*×3^ within a fixed Laplacian eigenbasis *U* ℝ ^*V×B*^, yielding time-varying meshes, ℳ^*t*^ = ℳ_0_ + *U* · *φ*^*t*^. This compact representation yields smooth, anatomically coherent deformations over time. To align meshes with observed image evidence, each ℳ^*t*^ is projected into radial space via DVS [35], producing ${\hat{I}}_{\theta_{s}}^{t}=\text{DVS}\left({ℳ}^{t}\right).$. The final training objective combines this supervision with geometric regularization terms [18], as in (11).

(11) $$\begin{array}{c} {ℒ}_{\text{mesh}}=\lambda_{1}\cdot {ℒ}_{\text{dice}}\left(I_{\theta_{s}}^{t},{\hat{I}}_{\theta_{s}}^{t}\right)+\lambda_{2}\cdot {ℒ}_{\text{lap}}\\ +\lambda_{3}\cdot {ℒ}_{\text{norm}}+\lambda_{4}\cdot {ℒ}_{\text{thick}}. \end{array}$$

where $I_{\theta_{s}}^{t}$ denotes slices in $M_{0}^{t}.{ℒ}_{lab},{ℒ}_{\text{norm}},{ℒ}_{\text{thick}}$ enforce mesh smoothness, normal consistency, and physiological wall thickness [18]. This formulation enables weak supervision, yielding temporally coherent 3D reconstruction directly from radial segmentations. *λ*_1-4_ are weighting factors, set to 1.0, 0.01, 0.01, and 0.01, respectively, based on a random search tuned to balance the relative magnitudes of the loss terms.

## IV Datasets and Experimental Setup

### A Our FeEcho4D Dataset

The dataset comprises fetal 4D echocardiography sequences acquired over a range of gestational ages and includes both healthy and congenital CHD cases, for a total of 52 annotated subjects. Among the CHD cases, Tetralogy of Fallot (ToF) and dextro-transposition of the great arteries (d-TGA) are represented. As summarized in Table II, the dataset provides spatiotemporal annotations of LV and its MYO and corresponding 3D meshes over time (4D). Annotation demonstrations and biomarker estimation examples are available at https://feecho4d.github.io/Website/. All experiments are conducted with patient-level data splitting to avoid subject overlap across training, validation, and test sets, and a stratified subset of subjects is reserved for testing. In addition, we will provide 50 additional unannotated 4D subjects to support semi-supervised and future methodological research.

### B Public MITEA Dataset

To evaluate the cross-dataset and cross-distribution generalizability (cross-age, i.e., fetal-to-adult) from radial slicing to reconstruction via segmentation, we additionally employ the adult echocardiographic MITEA dataset [17]. This dataset comprises 536 volumes from 134 subjects with a training–validation vs. testing split (number of volumes) as follows: healthy (304/24), LV hypertrophy (48/8), amyloidosis (36/12), aortic regurgitation (32/8), hypertrophic cardiomyopathy (28/4), dilated cardiomyopathy (20/4), and transplant (4/4).

### C Setup and Evaluation

All experiments were conducted using PyTorch and MONAI on an Ubuntu system equipped with four NVIDIA^QR^ RTX 3090 Ti GPUs (24 GB each), an Intel^QR^ Xeon W-2295 CPU (18 cores @ 3.00 GHz), and 128 GB RAM. All segmentation models were trained for 100 epochs with a batch size of 4 using the Adam optimizer (learning rate: 10^−4^), for both 256^2^ radial slices and 128^3^ volumetric inputs. Model-specific hyperparameters for UNet [8], SAM [13], MedSAM [14], I^2^UNet [11], and UNETR++ [10] were adopted from their respective publications, and all models were trained under the same experimental configuration as the proposed SCOPE-Net.

To automatically generate LV bounding-box prompts, we train a lightweight YOLO [39]-inspired detector (inference time: 7 ms per frame) using an ImageNet-pretrained ResNet-18 backbone [40], followed by global average pooling and a fully connected layer that predicts a normalized bounding box (*c_x_, c_y_, w, h*) ∈ [0, 1]^4^ per slice. The regression objective combines an 𝓁_1_ loss on box parameters with an IoU term, ${ℒ}_{\text{box}}=\|b-\hat{b}{\|}_{1}+(1-\text{IoU}(b,\hat{b})),$ where *b* and $\hat{b}$ denote the ground-truth and predicted boxes, respectively. The training parameters and optimizer are identical to those used for SCOPE-Net. Ground-truth bounding-box prompts are generated during MYO annotation by expanding the object bounds by at least 10% in each direction. Scribble prompts are synthesized by perturbing the object skeleton with randomized offsets and partial suppression to mimic realistic weak annotations. Both prompt types (see example in Fig. 8) are released with FeEcho4D to ensure full reproducibility.

3D mesh reconstruction was performed using GHD with 49 (7 × 7) Laplacian eigenmodes over 500 iterations, using 30k points per step and a learning rate of 10^−2^. Laplacian trade-off weights were set to: cotlap = 0.1, dislap = 1.0, stdlap = 0.1 [18]. As reconstruction baselines, we implemented voxel-based surface extraction using Marching Cubes [19] and correspondence-based non-rigid registration using NrCPD [20] and NrICP [21]. Given a binary volumetric segmentation $M_{\theta }^{t}\in {\left\{0,1\right\}}^{S\times H\times W}$, an isosurface $𝒮=\{x|M_{\theta }^{t}(x)=\tau \}$ is extracted using Marching Cubes at *τ* = 0.5, with optional 3D morphological regularization. For correspondence-based reconstruction, a canonical template ℳ_0_ ∈ ℝ ^*V*×3^ (identical to that used in GHD) is aligned to a target point cloud $P_{\text{MYO}}^{t},P_{\text{BG}}^{t}\in {\mathbb{R}}^{N\times 3}$ extracted from radial masks. NrCPD estimates a smooth non-rigid deformation *T* (*Y*) = ℳ_0_ + *GW*, where $G_{ij}=\exp\left(-||y_{i}-y_{j}|{|}_{2}^{2}/\left(2\beta^{2}\right)\right)$ enforces motion coherence (with *β* = 3.0) and *W* are deformation weights regularized by a smoothness parameter *α* = 5.0, optimized via EM for up to 150 iterations (tolerance 10^−3^, outlier weight *w* = 0). NrICP first computes a global rigid alignment (*R, t*) by minimizing $\sum_{i}||Ry_{i}+t-x_{c(i)}|{|}_{2}^{2}$ over nearest-neighbor correspondences, using up to 10 ICP iterations with convergence tolerance 10^−6^, followed by non-rigid refinement using a regularized least-squares formulation with edge-length constraints and a coarse-to-fine stiffness schedule *α* ∈ {5.0, 2.0, 1.0, 0.5, 0.1}, optimized for up to 30 iterations per stiffness level. In both cases, the deformed template vertices with fixed connectivity define the reconstructed surface.

Endocardial (ENDO) and epicardial (EPI) segmentations are gauged for overlap accuracy via the Dice similarity coefficient (DSC), boundary robustness via the 95th percentile Hausdorff distance (HD95) and mean average surface distance (MASD), and disconnected region artifacts via percentage island area (PIA) [30]. Reconstruction quality is evaluated using the 3D DSC and Chamfer distance (CD), which measures the average bidirectional surface distance between the predicted and ground-truth meshes. Functional accuracy is further assessed via correlation of stroke volume (*V*_stroke_), EF, and GLS.

## V Experimental Results

### A Radial Slicing for 3D LV Reconstruction

Table III compares segmentation accuracy and downstream 3D reconstruction performance on the MITEA dataset using SAX, LAX, radial slicing, and direct 3D volumetric segmentation. Across UNet, SAM, and MedSAM (fine-tuned) backbones, radial slicing achieves the highest slice-wise 2D DSC in all cases (last column), indicating more consistent per-slice segmentation than Cartesian views and 3D volumes. This improvement is attributable to the standardized *U-shaped* myocardial appearance induced by our novel radial sampling, which reduces inter-slice variability present in SAX and LAX views, as further evidenced by higher intra-patient inter-slice correlation for radial slices (78%) compared with LAX (64%) and SAX (44%).

Following 3D reconstruction, radial views consistently yield superior 3D reconstruction accuracy, with higher 3D DSC and lower surface distance metrics than SAX, LAX, and volumetric segmentation in nearly all statistically significant comparisons (*p* < 0.05, paired t-test). This indicates that radial segmentation yields improved 3D reconstruction with greater anatomical fidelity and robustness. The improvement from SAX and LAX is likely due to higher-quality 2D segmentations in image slices, whereas the reduced performance of direct 3D volumetric segmentation may be attributed to its higher complexity and weaker boundary delineation. Reconstructing 3D meshes from sparse radial 2.5D segmentations also provides improved computational efficiency, requiring substantially fewer FLOPs per frame than 3D volumetric approaches (51 *G* vs. 79 *G*).

### B SCOPE-Net on Radial Views and Ablation Studies

**Fig. 4:**
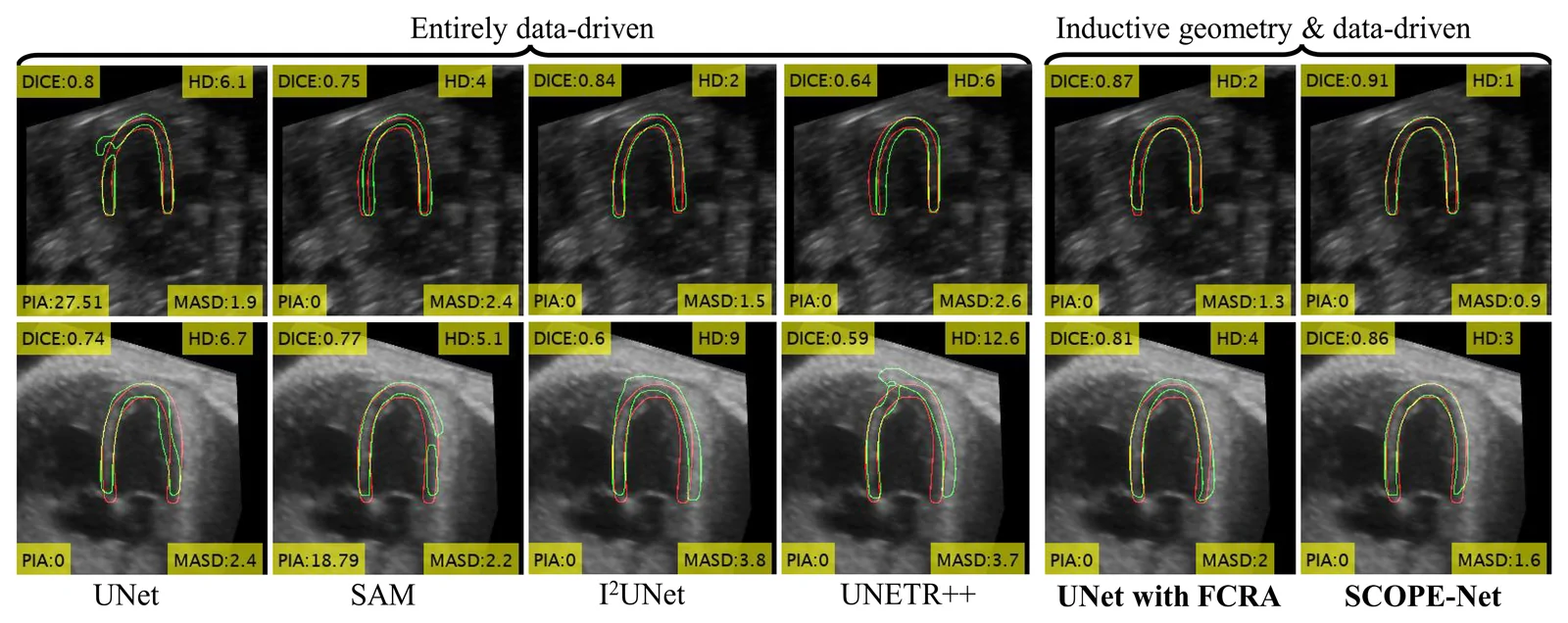
Qualitative segmentation comparison between purely data-driven methods (UNet, SAM) and geometry-aware approaches (UNet+FCRA, SCOPE-Net) on the FeEcho4D dataset. Ground-truth MYO contours (red) and predictions (green) are overlaid, showing improved boundary alignment and anatomical consistency for geometry-aware models.

Table IV presents quantitative segmentation results on the FeEcho4D and public MITEA datasets, comparing the proposed SCOPE-Net with strong purely data-driven baselines, including UNet, SAM, I^2^UNet, and UNETR++. Across both fetal and adult datasets, SCOPE-Net consistently achieves the best performance in terms of boundary accuracy (HD95, MASD), overlap (DSC), and robustness to outlier regions (PIA), with nearly all improvements reaching statistical significance (*p* < 0.05, paired t-test). These gains are particularly evident in challenging anatomical regions such as the LV apex and lateral wall, where competing methods exhibit boundary fragmentation and isolated predictions, as shown in Fig. 4, indicating improved anatomical plausibility of SCOPE-Net segmentations.

While UNETR++ and I^2^UNet are state-of-the-art models originally developed for Cartesian 3D medical images, their adaptation to 2D radial echocardiographic slices remains slice-wise, with attention and feature fusion confined to intra-slice processing and without equivariance to the inherent radial symmetry *I*_*θ*_ ≈ flip(*I*_*π*−*θ*_). In contrast, SCOPE-Net explicitly enforces angular flip-equivariance in feature coupling via the proposed FCRA module and representation-level consistency constraints via ISAI, thereby suppressing echo-specific topological errors, such as boundary fragmentation and isolated islands. As reflected by consistently lower HD95, MASD, and PIA across both datasets, SCOPE-Net produces smoother and more contiguous myocardial contours while remaining computationally efficient (56 *G* FLOPs/frame vs. 79 *G* in 3D volume), making it suitable for large-scale 4D echocardio-graphic analysis.

#### 1) Ablation Studies

SCOPE-Net’s superiority arises from three core innovations: (1) a geometry-adaptive encoder equipped with FCRA, (2) training with the ISAI-driven SISD framework for symmetry-consistent representation learning, and (3) prompt-aware feature modulation. The contribution of each component is evaluated in Table IV on the FeEcho4D dataset. Incorporating FCRA refines a vanilla UNet by reducing false positives and disconnected regions, thereby encouraging single-connected MYO contours, while the SISD framework further exploits angular symmetry prior to improving cross-angular view consistency, resulting in enhanced robustness as reflected by reduced PIA (shown in Fig. 4). Prompt-guided modulation complements these geometry-aware components by steering the network toward clinically plausible shapes, facilitating more reliable downstream 3D reconstruction from sparse radial 2D slices via GHD (see Table V).

Beyond these gains, feature visualizations in Fig. 6 (left column) reveal clear representational differences across models. The data-driven UNet learns diffuse, weakly localized features with substantial color mixing, suggesting entangled representations and poor separation between the LV cavity and MYO. Incorporating prompts via SAM improves localization but still yields fragmented and spatially inconsistent representations along the MYO wall. In contrast, SCOPE-Net (and its constituents, FCRA and SISD) produces spatially coherent features with distinct responses aligned with the LV cavity and MYO regions, indicating the emergence of disentangled, anatomy-aware latent representations rather than merely improved pixel-wise mask predictions.

The correlation analysis in Fig. 6 (right column) further provides mechanistic insight. The angular symmetry observable in the input images is only weakly preserved by purely data-driven encoders (middle plot), whereas SCOPE-Net explicitly embeds this acquisition-induced symmetry in the latent space, thereby better preserving angularly symmetric correlations. This enables the suppression of slice-wise noise and topological errors, resulting in a substantial reduction in PIA and improved boundary accuracy (Fig. 4).

#### 2) Sensitivity to LV Axis and Rotation Center

**Fig. 5:**
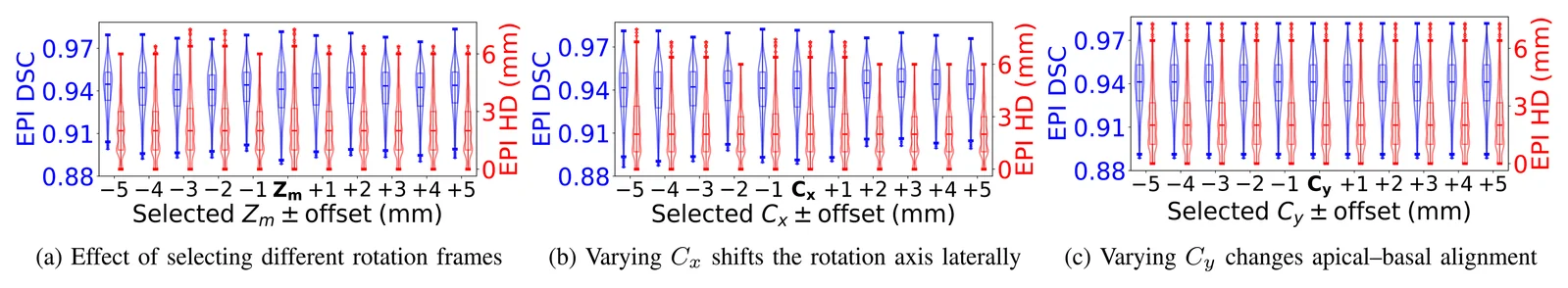
Effect of rotation-frame and rotation-center selection on radial slice extraction.

**Fig. 6:**
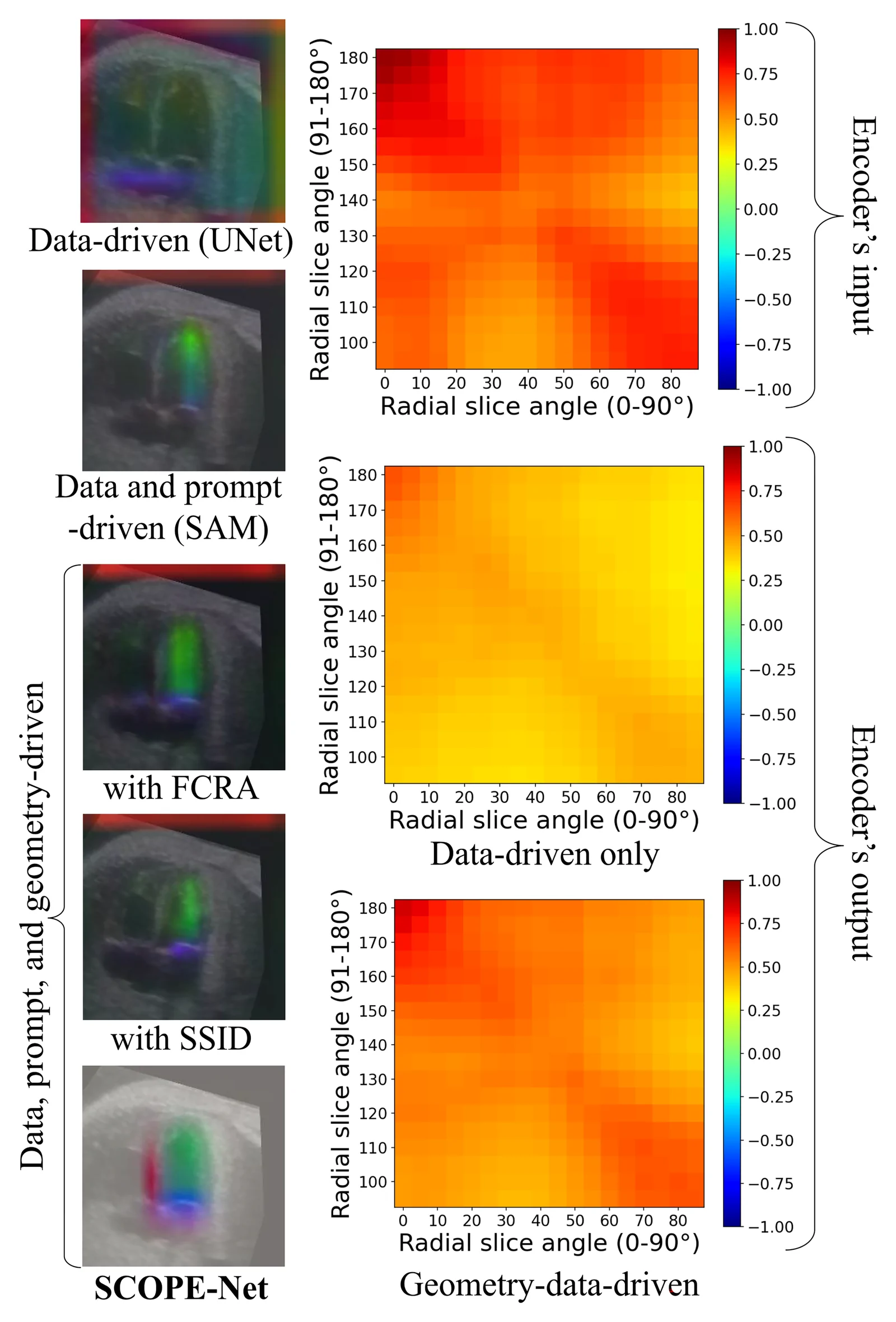
**Left column:** feature maps overlaid on the images, with PCA-projected embeddings mapped to RGB. **Right column:** the correlation between angularly symmetric slice pairs (*I*_*θ*_, *I*_*π−θ*_) for both the input images and the corresponding encoded representations, w/ and w/o geometry-aware learning.

To assess the effect of poor specification of LV rotational axis, we performed a sensitivity analysis by perturbing the coordinates of the LV center (in plane: *C_x_, C_y_*, out-of-plane: *Z_m_*) by ±5 mm and evaluating epicardial DSC and HD95. As shown in Fig. 5, perturbations of *Z_m_* and *C_x_* result in non-significant performance changes (*p* > 0.05) with small effect sizes (Cohen’s *d* ≈ 0.2), while apical–basal perturbations (*C_y_*) produce identical performance across the tested range, suggesting very little effect. This suggests limited effects of poor LV rotational axis specification.

In addition, the LV rotational axis can be automatically estimated from the LV bounding box using a lightweight YOLO-based detector (≈ 7 ms/frame) (see Section IV-C), yielding an average inter-frame error of ≈ 4.5 mm. This error lies within the evaluated tolerance range (±5 mm in Fig. 5), indicating that such automated estimation is sufficient in practice under frame-to-frame motion and high fetal heart rates.

#### 3) Prompt Robustness Analysis

Fig. 8 evaluates the effect of different prompt modalities on FeEcho4D, including manual and automatic bounding boxes and manual scribble prompts. Automatic bounding boxes are generated using our pre-trained lightweight network (7 ms inference, ≈ 5 mm average error) (details in Section IV-C). All prompt-conditioned variants significantly outperform the baseline UNet, while no statistically significant differences are observed across prompt types, indicating robustness to prompt source and granularity. Echo images are often obscured by noise and signal losses, and prompts therefore act as coarse localization cues to mitigate this. The comparable performance achieved with automatically generated prompts further demonstrates that the proposed framework works well with fully automatic inference and does not strictly require manual interaction.

#### 4) Disease Subgroup Analysis

**Fig. 7:**
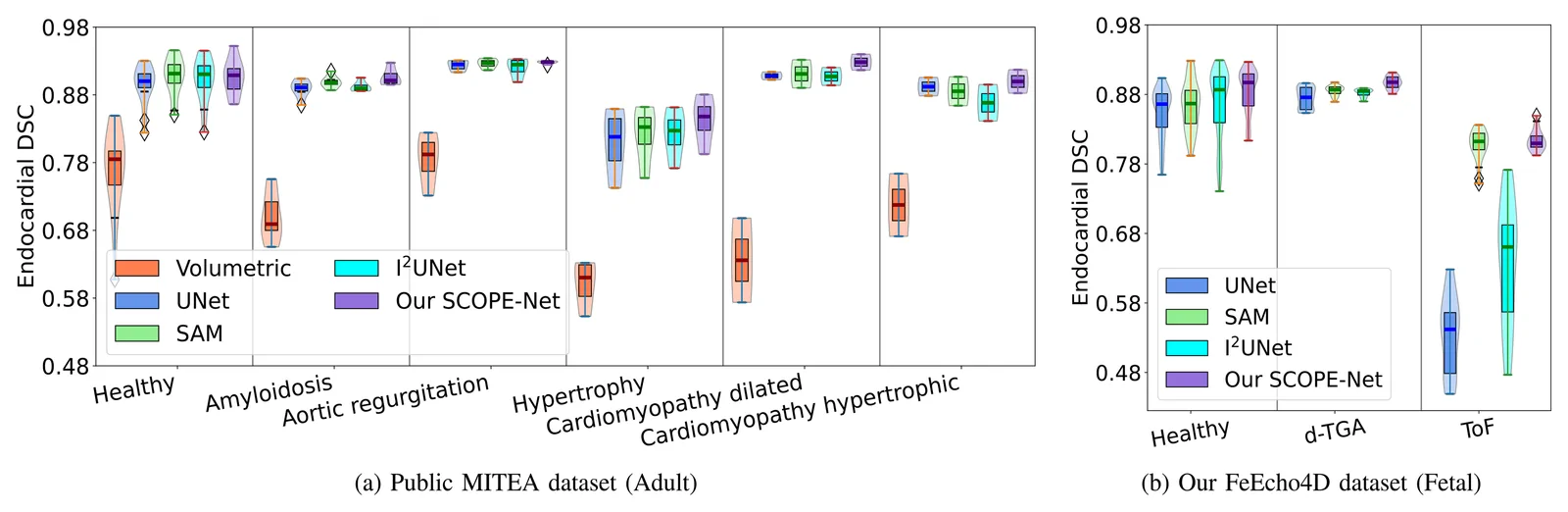
Segmentation performance across cardiac disease subgroups, comparing volumetric versus radial views and purely data-driven models (UNet, SAM, and I^2^UNet) against the proposed geometry-aware SCOPE-Net.

**Fig. 8:**
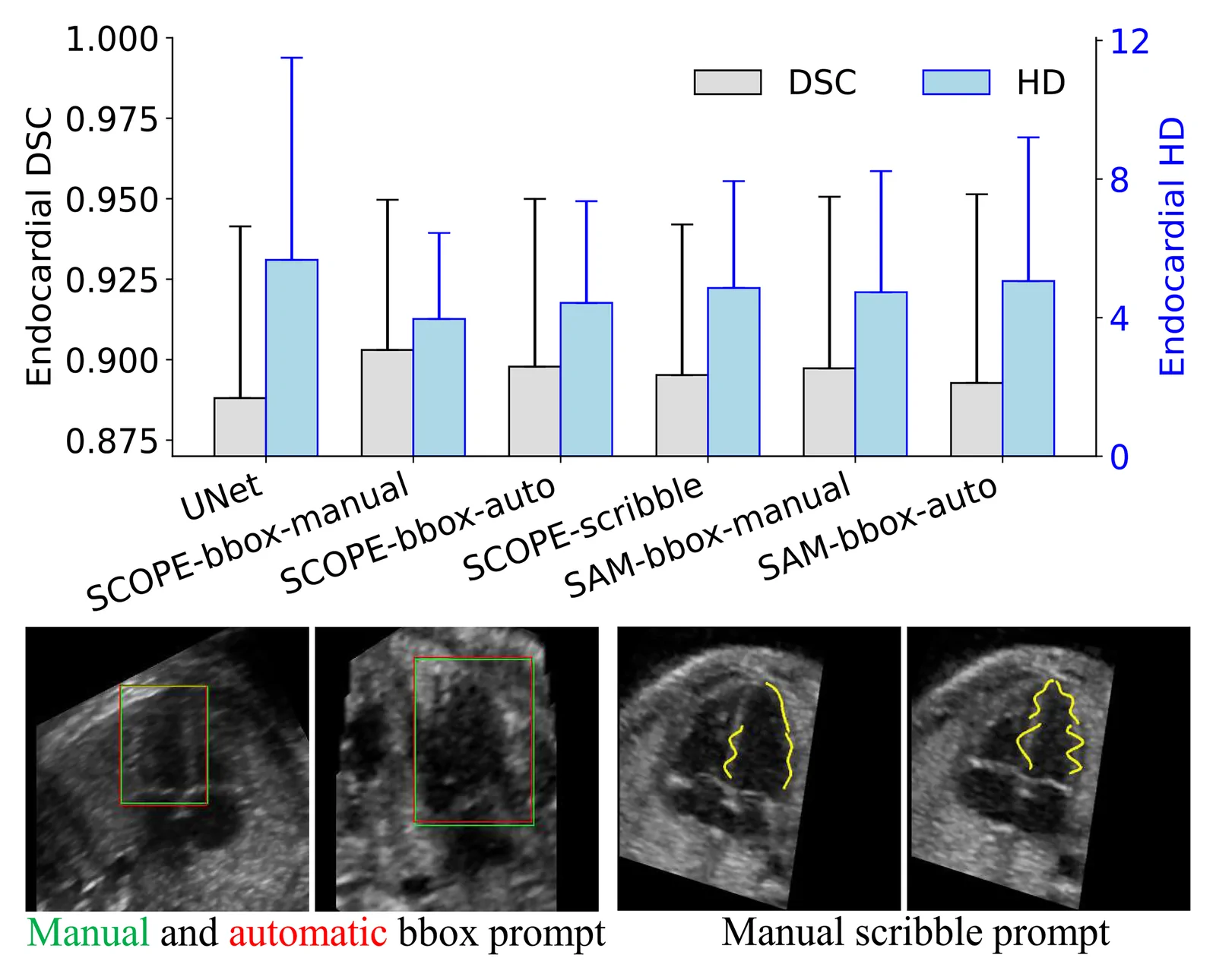
Effect of prompt type on segmentation performance on the FeEcho4D dataset, showing robustness of SCOPE-Net and SAM to different prompt modalities.

Fig. 7 summarizes segmentation performance across cardiac disease subgroups for both the public MITEA and our FeEcho4D datasets. Across diverse cardiac pathologies in both datasets, SCOPE-Net consistently achieves the highest endocardial DSC with reduced variance compared with purely data-driven baselines, indicating improved robustness. Volumetric segmentation remains consistently weaker than radial slicing, likely due to the absence of explicit anatomical priors and constraints. These findings indicate that the proposed geometry-aware framework generalizes effectively across age groups (adult and fetal) and clinical conditions, maintaining robustness under varying anatomical and imaging challenges.

Performance nonetheless varies across disease subgroups. In adults, hypertrophic phenotypes remain the most challenging due to reduced cavity motion, hyperdynamic contractions, and poor lateral wall visibility, resulting in lower accuracy across all methods, particularly volumetric approaches. In the fetal cohort, increased variability is also observed in ToF cases, particularly in purely data-driven models, primarily due to some asymmetry in ventricular geometry, reduced LV dominance, and frequent acoustic shadowing in fetal echo. Nonetheless, our geometry-aware framework demonstrated an ability to remain effective even with some anatomical asymmetry.

### C 3D Reconstruction and Cardiac Biomarker Estimation

We evaluate the clinical validity of the proposed framework by estimating EF and GLS from radial segmentation followed by surface mesh reconstruction and compare the results against different segmentation and reconstruction methods, as presented in Table V and Fig. 10. For 2D EF estimation, EDV and ESV were obtained by dividing the segmented LV cavity into 50 long-axis segments, assuming circular cross-sections, and integrating disc areas via Simpson’s rule. GLS was computed from the epicardial mesh by measuring apex-base distances across 30 radial long-axis planes, trimming the five longest and shortest values, and averaging the remaining lengths to quantify longitudinal shortening.

**Fig. 9:**
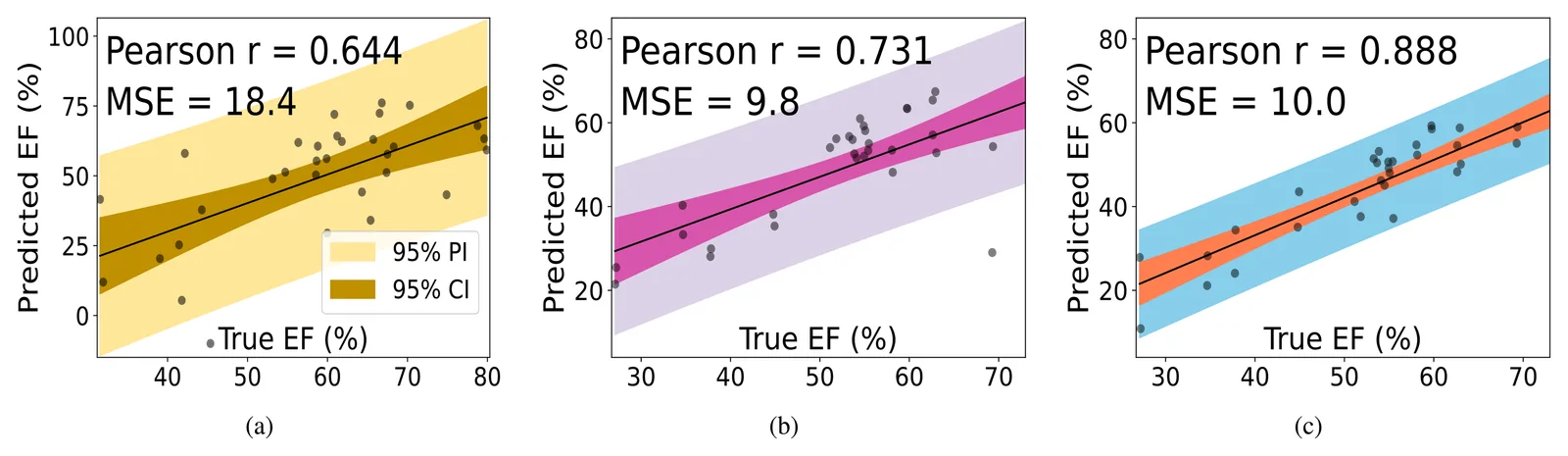
EF estimation from (a) UNet2D (LAX) + Simpson’s, (b) UNet3D, and (c) SCOPE-Net + GHD on the MITEA dataset. PI and CI denote prediction and confidence intervals.

In two subjects with cardiac amyloidosis, the 2D Simpson’s method yielded physiologically implausible measurements, showing negative EF values (ESV > EDV) and abnormally low EF (see Fig. 9), arising from asymmetric wall thickening and Simpson’s inherent underestimation of EDV in such pathology. These errors reflect the inability of a shape-assumption-based 2D approach to capture the complex ventricular morphology in amyloidosis. In contrast, as shown in Fig. 9 (b) and (c), 3D reconstruction from the proposed radial framework (SCOPE-Net+GHD), as well as direct volumetric segmentation, yields accurate ventricular volume estimates in both cases and improves overall measurement precision across the test cohort. This highlights the advantage of true 3D quantification over 2D measurements that rely on simplified assumptions about cardiac shape (see Table V).

**Fig. 10:**
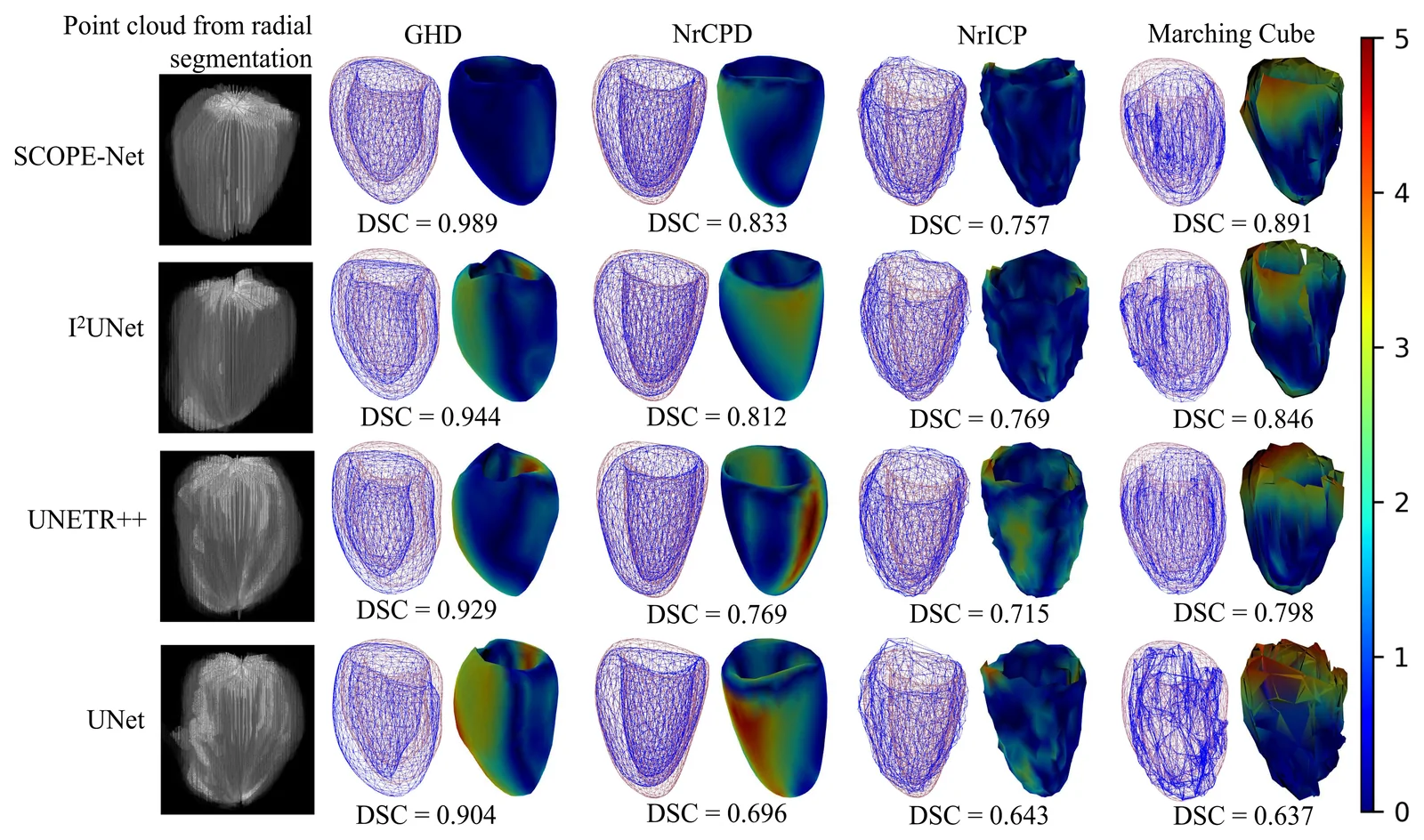
3D surface reconstruction (ground-truth: red wireframe and estimated: blue wireframe) from point clouds (left) derived from radial segmentation on the MITEA dataset. Surface color maps indicate point-to-surface error (mm). Incorporating the radial inductive bias (Section III-B1), SCOPE-Net yields more coherent point distributions, improved reconstruction accuracy, and more reliable cardiac biomarker estimation (see Table V) compared to baseline methods.

These results in Table V and Fig. 10 underscore two key insights. First, although UNet3D benefits from full volumetric supervision, which is cumbersome to achieve in 3D echocardiography, especially in fetal 3D, it suffers from poor elevational resolution, lateral wall ambiguity, and temporal inconsistencies, limiting its reliability in functional estimation. Second, while annotation-efficient radial UNet, I^2^UNet, UNETR++, and SAM offer superior segmentation metrics (e.g., DSC, HD95), they produce disconnected or anatomically implausible MYO contours (see Fig. 4), particularly near the apex and lateral wall. These discontinuities result in sparse or fragmented point clouds, which degrade the reconstructions by introducing mesh warping artifacts (see Fig. 10) that impair biomarker accuracy. SCOPE-Net’s embedding of symmetry-aware anatomical priors enables the generation of contiguous and topologically faithful MYO contours. These improvements manifest as substantially lower PIA scores and denser, anatomically valid point clouds, which, in turn, facilitate smooth, coherent 3D mesh reconstruction, as shown in Fig. 10. The resulting surfaces preserve spatial integrity over time (as shown in the 4D video at https://feecho4d.github.io/Website/), supporting robust estimation of clinically relevant biomarkers. This end-to-end consistency from segmentation to functional quantification shows the value of symmetryaware radial modeling for accurate, label-efficient 4D cardiac analysis, even in anatomically ambiguous or artifact-prone imaging scenarios.

As shown in Table V, voxel-based reconstruction via Marching Cubes achieves competitive surface metrics, often outperforming correspondence-based non-rigid registration (NrICP and NrCPD), as it directly extracts an iso-surface that closely follows the segmented radial slices. However, Fig. 10 shows that this voxel-to-surface conversion introduces discretization artifacts and locally irregular surface texture. NrCPD yields visually smoother surfaces through strong motion-coherence regularization, but this smoothness is driven primarily by the template deformation model rather than by strict adherence to the segmentation-derived geometry, resulting in reduced geometric accuracy. NrICP, which relies on hard point-to-point correspondences, performs poorly under radial sampling, where non-uniform point density and correspondence ambiguity lead to unstable deformations, increased surface error, and degraded surface quality (see Fig. 10). Overall, these results reveal a trade-off between metric fidelity and surface regularity. GHD resolves this by modeling deformation in a low-dimensional spectral space defined by the mesh Laplacian, which enforces global smoothness while remaining tightly coupled to the segmentation-derived geometry, yielding anatomically coherent LV surfaces under sparse radial supervision. This is consistent with our previous studies of GHD [18].

**Fig. 11:**
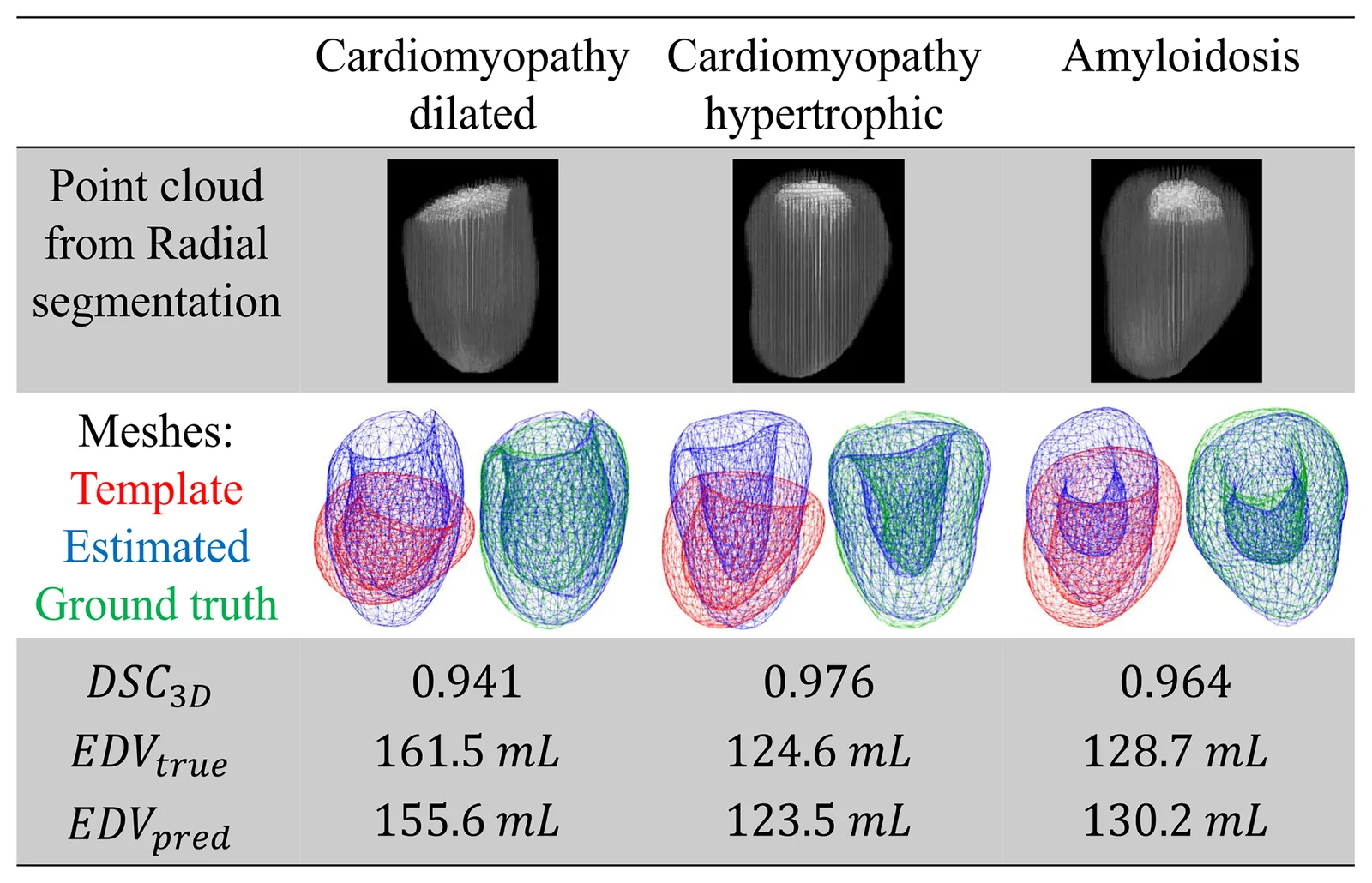
GHD reconstruction on pathological cases in the MITEA dataset, demonstrating accurate recovery of localized geometric variations and close agreement with ground truth, without bias toward a smooth template.

To assess the representation capacity of GHD, we evaluated pathological cases with non-trivial geometric variations, including dilated and hypertrophic cardiomyopathy and amyloidosis in the MITEA dataset. As shown in Fig. 11, the reconstructed meshes closely match the ground truth and preserve localized disease-specific features, such as asymmetric wall thickening and non-uniform morphology, rather than converging to a smooth template. This is enabled by deformation guided by segmentation-derived 3D point clouds from radial views, allowing adaptation to case-specific anatomy. The proposed framework shows strong correlation agreement with reference 3D volumes (EF: 88.8%, GLS: 91.1%). These results indicate that, despite operating in a low-frequency Laplacian subspace, the deformation remains sufficiently expressive, with such variations well captured within the dominant modes of cardiac shape variation, consistent with our prior work [18].

## VI Discussion

This work introduces a full-stack framework for 4D reconstruction of fetal heart geometry from 4D echocardiography, combining a novel 2.5D radial segmentation with Graph-Fourier–based mesh reconstruction for downstream biomarker estimation. By leveraging the structured geometric nature of radial acquisition, our framework achieves improved segmentation accuracy, enhanced anatomical fidelity in 3D meshes, and more accurate estimation of clinical biomarkers, all while reducing reliance on dense volumetric annotations. Unlike conventional data-driven approaches, our method explicitly embeds anatomical priors, specifically acquisition-aware inductive bias, into both the network architecture and training objectives. This integration leads to gains not only in segmentation performance but also in anatomical plausibility and generalizability in 4D heart reconstruction across varied imaging conditions and disease types.

By establishing an automatic, accurate, and computationally and annotation-efficient alternative to volumetric 3D analysis for 3D/4D reconstruction from 4D echocardiography, this work helps alleviate long-standing processing and visualization bottlenecks associated with 3D fetal imaging. The proposed framework enables practical access to the advantages of 3D echocardiography over conventional 2D analysis, providing more comprehensive anatomical coverage, reducing the risk of missing clinically relevant regions, and yielding more precise functional quantification without reliance on simplified geometric assumptions as in the 2D quantification.

### Radial slicing introduces a built-in anatomical prior, not just sampling

Unlike SAX or LAX views that rely on operator-defined planes, radial slicing ensures that each view consistently passes through the LV center, symmetrically capturing both septal and lateral walls. This acquisition geometry mitigates common challenges in ultrasound imaging, including acoustic shadowing and lateral wall dropout. Our framework leverages this structure not merely as a sampling strategy but as a geometric prior embedded throughout the pipeline from segmentation to reconstruction. Beyond simplifying annotation, this acquisition structure inherently guides feature learning by aligning with the heart’s physical symmetry. Rather than treating it as a preprocessing step, we embed this prior throughout the learning process, allowing the model to exploit spatial regularities that are otherwise absent in Cartesian views. This reparameterization of the data fundamentally changes where structure can be enforced in the pipeline.

### Representation–reconstruction decoupling as a design principle

A further aspect of this work is the explicit separation between representation and reconstruction. Conventional volumetric approaches typically require dense 3D ground-truth labels and rely on full-volumetric prediction followed by direct surface extraction, which is challenging to obtain in fetal echocardiography and is sensitive to local errors. In contrast, our pipeline first learns a structurally coherent, symmetry-aware radial representation, leveraging the fact that radial views produce a U-shaped MYO contour that is substantially easier and more consistent to annotate in 2D. Continuous 3D surfaces are then reconstructed via GHD in geometric space. The comparative results (Table V and Fig. 10) suggest that 4D reconstruction quality depends more on the anatomical consistency of this intermediate representation than on voxel density alone, turning surface recovery from an ill-conditioned post-processing step into a better-conditioned geometric optimization problem.

### SCOPE-Net turns radial geometry into an inductive advantage

At the core of SCOPE-Net is the FCRA module, which enforces angular symmetry by aggregating features across mirrored radial slices. Unlike conventional attention, FCRA operates in angular space and explicitly models reflectional equivariance, yielding anatomically consistent segmentations, especially in dropout-prone regions. To further exploit radial geometry, the SISD framework introduces a self-supervised objective (ISAI) that aligns features across independent augmentations, thereby improving representation robustness, unlike conventional student-teacher models. Prompt-based cues, such as bounding boxes or scribbles, serve as soft spatial priors, refining predictions in ambiguous cases while preserving inference efficiency. These components collectively transform radial acquisition from a sampling choice into a structural prior, guiding both the architecture and training of SCOPE-Net for anatomically faithful, controllable, and label-efficient segmentation in echocardiography.

### Anatomical continuity is critical for both reconstruction and biomarkers—ultimately, it is topology that makes reconstruction possible

While segmentation accuracy is often measured using overlap metrics like the DSC score, we demonstrate that structural consistency, such as smooth contours, reduced fragmentation, and uniform wall thickness, is essential for high-quality 3D reconstruction. Fragmented or disconnected masks, even with high DSC, generate noisy point clouds that compromise mesh fitting and impair the estimation of clinical biomarkers. In contrast, our symmetry-aware SCOPE-Net produces anatomically coherent segmentations that yield smoother 3D surfaces and denser point clouds, enabling more accurate estimation of EF and GLS. These improvements are validated by higher correlations with clinical ground truth and visibly improved mesh topology (Fig. 10).

### FeEcho4D as a community resource

Beyond the proposed framework, FeEcho4D serves as a methodological resource for 4D echocardiography research. To our knowledge, it is the first publicly available fetal dataset providing temporally coherent 3D meshes with standardized radial views across healthy and CHD cases, complemented by the adult MITEA dataset for cross-age and cross-distribution evaluations. The combination of dense annotations and additional unannotated 4D studies supports research on semi-supervised learning, domain adaptation, and topology-aware segmentation and reconstruction, enabling data-efficient and anatomically constrained 4D cardiac analysis.

## VII Limitations and Future Directions

**Fig. 12:**
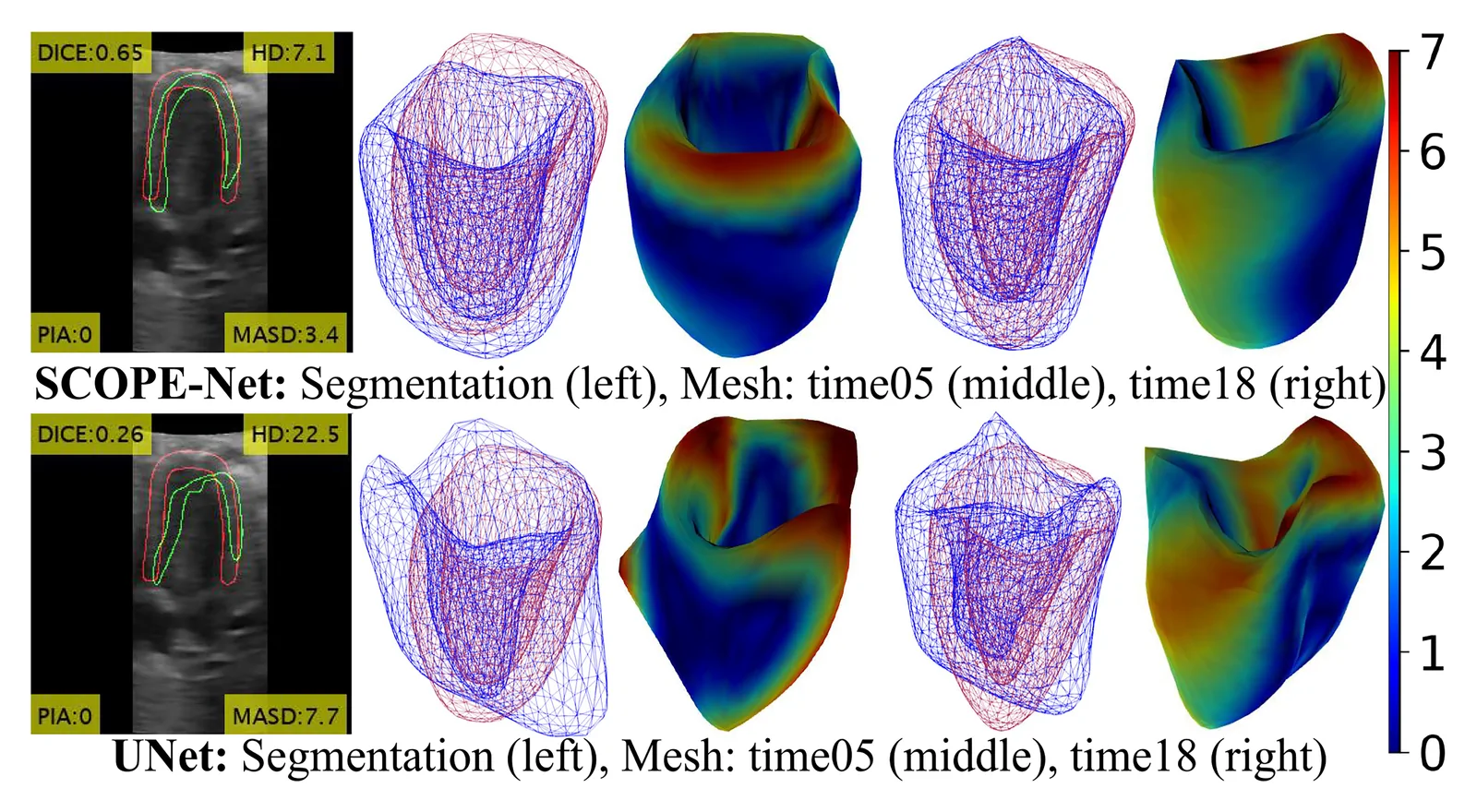
Example of poor results: segmentation-to-3D surface reconstruction on our FeEcho4D dataset. Surface color maps indicate point-to-surface error (mm). **In segmentation:** ground-truth MYO contours (red) and predictions (green) are overlaid on the image (left). **In 3D reconstruction:** ground-truth: red wireframe and estimated: blue wireframe.

A limitation of the proposed framework arises from through-plane sampling variability in fetal echocardiography, where slice counts vary substantially across subjects (≈40–100), resulting in reduced resolution in the through-plane/elevational axis of the 3D image. This anisotropy is most evident for near-orthogonal radial views (≈90°), where increased reliance on interpolation leads to blurred MYO boundaries and slight spatial shifts that propagate to 3D reconstruction (Fig. 12) (left). While SCOPE-Net preserves anatomically plausible shapes under these conditions, purely data-driven baselines (e.g., UNet) may produce non-physiological meshes (Fig. 12). Residual global shifts in extreme low-slice cases reflect fundamental acquisition limitations rather than deficiencies of the proposed method. Future work may incorporate slice-density-aware confidence weighting or joint segmentation-reconstruction optimization to mitigate sparse through-plane sampling, while recognizing that acquisition density remains the primary limiting factor.

**Fig. 13:**
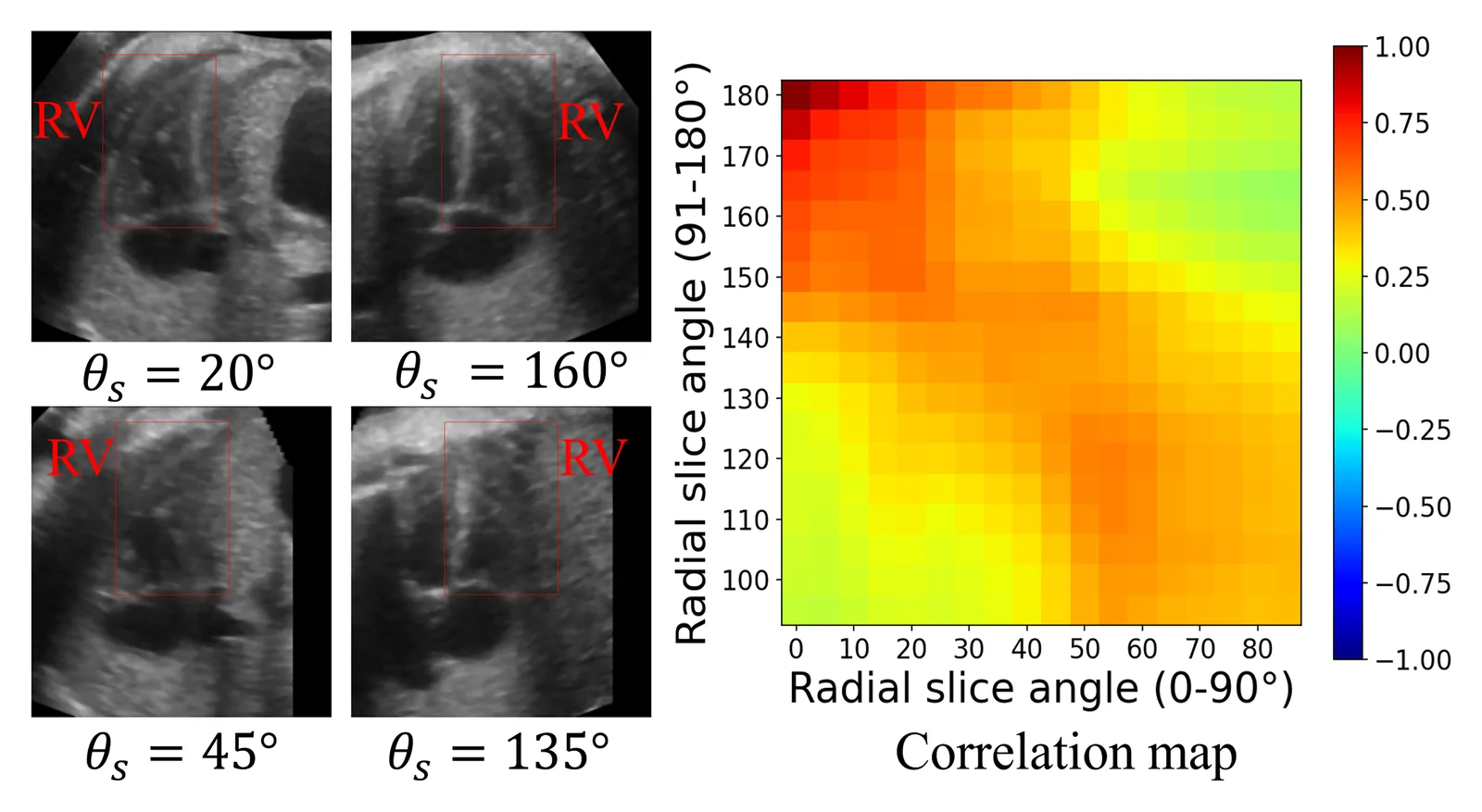
Radial symmetry in the RV. Angularly paired RV slices (*θ* and π − *θ*), and their correlation map exhibit a clear diagonal structure, indicating approximate mirror symmetry in the RV and supporting the applicability of the radial formulation beyond the LV.

While this work focuses on the LV, fetal physiology yields comparable pressures and geometry in both ventricles, resulting in an approximately straight septum and near-bilateral morphology [41]. Consequently, the proposed radial formulation is applicable to the RV and enables biventricular reconstruction. Fig. 13 substantiates this by showing angularly paired RV slices and a clear diagonal correlation structure, indicating approximate mirror symmetry in the RV as well. These results suggest that the acquisition-induced symmetry exploited by our framework is not LV-specific in the fetal heart. Extending the framework to full biventricular modeling, atrial structures, or anatomy with weaker symmetry will require adaptive pairing and asymmetry-aware learning, which we identify as an important direction for future work.

Although the radial flip assumption holds across all study cases and underlies the consistent gains of SCOPE-Net across disease categories (Fig. 7), it may be violated in cases of severe anatomical asymmetry or imperfect axis definition. Future work will therefore explore dynamic or data-driven slice pairing and fully automatic axis estimation (e.g., via landmark regression or lightweight detectors) to remove manual intervention and further improve robustness. Expanding prompt modalities beyond bounding boxes and scribbles to include anatomical landmarks or text guidance may enhance interaction. Beyond fetal cardiology, the proposed segmentation-to-4D-reconstruction framework could extend to other radially acquired modalities, such as 4D CT tumor tracking or cine MRI motion analysis, with additional directions including parameter-efficient integration with large pretrained models and unified multi-task learning for segmentation, reconstruction, and biomarker estimation.

## VIII Conclusion

We address key challenges in 4D fetal echocardiography by introducing FeEcho4D, the first benchmark dataset with dense 4D annotations, and by proposing a full-stack framework that integrates segmentation, reconstruction, and biomarker estimation within an acquisition-aware design. By integrating radial slicing, symmetry-aware learning, and graph-based reconstruction, our approach generates anatomically coherent 4D meshes from sparse 2D annotations, significantly reducing reliance on full 3D labels. Embedding geometric inductive biases improves accuracy, efficiency, and clinical relevance, achieving high agreement with ground-truth biomarkers (EF: 0.888; GLS: 0.911) and outperforming both 2D + Simpson’s method and volumetric 3D segmentation. This acquisition-driven pipeline offers a scalable, annotation-efficient, and clinically interpretable solution for fetal cardiac analysis, with potential applicability to other modalities where radial views can be acquired or synthesized.

## Figures and Tables

**Table I:** Summary of Our Contributions Along Four Key Aspects Compared to Representative Prior Work

Our Contributions	Prior Work
**FeEcho4D Dataset:** We introduce FeEcho4D, the first publicly available benchmark for 4D fetal echocardiography with full 3D+time LV coverage, comprising 52 annotated subjects. Volumes are annotated via a novel radial slicing strategy, enabling consistent 4D annotation and robust downstream analysis. In addition, we will provide 50 additional unannotated 4D studies to support semi-supervised and future methodological research.	Public fetal echocardiography datasets are limited to 2D frames or short cine clips with sparse spatial and temporal coverage. No public dataset provides full 4D (3D over time) fetal volumes (not even for adults) for volumetric learning and evaluation.
**Symmetry-Aware, Prompt-Guided Segmentation:** We propose SCOPE-Net (**S**ymmetry-**CO**nsistent, **P**rompt-**E**nhanced **N**etwork), a geometry-adaptive network that explicitly encodes the angular symmetry induced by radial acquisition as a geometric inductive prior via slice-to-slice symmetric equivariance. A bilateral, learnable FCRA module couples features across angularly symmetric slice pairs and adaptively regulates information ex-change based on feature consistency, suppressing cross-slice propagation when correspondence is weak or inconsistent. In addition, spatial prompts (e.g., boxes or scribbles) are injected via gated feature modulation to support robust, interactive refinement.	Most existing models (UNet [8], nnUNet [9], UNETR++ [10], I^2^UNet [11], BATNet [12], SAM [13], MedSAM [14]) operate on Cartesian SAX/LAX views or full volumes and are purely data-driven, without explicitly encoding geometry or anatomical symme-try, leading to inter-slice inconsistencies and anatomically implausible structures, and un-stable 4D reconstructions, particularly under sparse or anisotropic sampling.
**Symmetry-induced Self-distillation Training Framework:** We introduce a geometry-aware self-supervised learning scheme that exploits acquisition-induced symmetry for representation-level supervision. Our SISD frame-work, equipped with an ISAI objective, enforces feature consistency between symmetrically paired slices under independently sampled augmentations, enabling label-free self-distillation. SISD defines semantically grounded positive pairs directly from anatomical symmetry and is fully self-contained: its supervisory signal arises from the interaction between the symmetry-aware architecture and independent augmentations applied to each slice in a symmetry pair, requiring neither a teacher network nor a memory bank.	Existing training paradigms rely on full su-pervision at the output level [8]–[14] or generic SSL [15], [16], where positive pairs are defined via augmentations or external teachers rather than anatomy. Consequently, these methods lack a geometry-grounded, representation-level supervisory signal and depend on teacher networks or memory banks, limiting robustness under noise, signal dropout, and sparse, anisotropic sampling.
**Graph-harmonic 4D Reconstruction and Biomarker Estimation: We** develop a GHD module, combined with Differentiable Voxel Slicing (DVS), to reconstruct temporally coherent 4D LV meshes from sparse radial 2.5D segmentations. On both FeEcho4D and the public Ml’l’EA [17] datasets, this integrated reconstruction pipeline yields smoother, anatomically consistent meshes and significantly improves downstream estimation of clinically relevant biomarkers such as EF and Global Longitudinal Strain (GLS).	3D reconstruction via voxel-based Marching Cubes directly discretizes volumetric labels and yields jagged, topology-unstable surfaces under sparse or anisotropic sampling, while template morphing-based NrCPD or NrlCP relies on dense correspondences and becomes brittle under noisy or sparse masks.

**FCRA:** Flip Consistent Radial Attention, **SISD:** Self-induced Self-destillation, **ISAI:** Inter-Slice Augmentation Invariance, **GHD:** Graph-Harmonic Deformation, **EF:** Ejection Fraction, **LV:** Left Verticle, **SAX:** Short Axis, **LAX:** Long Axis, **SSL:** Self-supervised Learning, **NrCPD:** Non-rigid Coherent Point Drift, and **NrICP:** Non-rigid Iterative Closest Point.

**Table II:** Summary of the Feecho4D Dataset, Including Clinical Composition, Data Split, and Acquisition Settings. Percentages (%) Indicate the Proportion of 3D Volumes (Or 2D Radial Slices) Within Each Class Assigned to the Corresponding Split

Category	Description	Details			
**Clinical Composition**	Healthy	**4D subjects:** 47, **3D volumes:** 1684, **2D radial slices:** 62308			
	ToF	**4D subjects:** 2, **3D volumes:** 47, **2D radial slices:** 1739			
	dTGA	**4D subjects:** 3, **3D volumes:** 114, **2D radial slices:** 4218			
**Gestational Age** **Acquisition Mode**	Range (weeks)	24–32			
	Imaging mode	eSTIC 4D (single trimester acquisition, highest quality setting, 50−70° sweep)			
	Scanner models	GE Vbluson Expert 22 (eM6CG3), Expert 10 (RM6C)			
	Export software	4DView (GE Healthcare)			
**Ethics**	Approval ID	1009/2017 (Kepler University Hospital)			
	Consent	Informed consent obtained			
**Resources**	Project website	https://feecho4d.github.io/Website/			
	Source codes	https://github.com/kamruleee51/FeEcho4D			
			**Class**	**3D Volumes**	**2D Radial Slices**
**Patient-level splits**	**Training (42 subjects)**		Healthy (40 subjects)	1 1418 (84.2%)	52466 (84.2%)
			ToF (1 subject)	26 (55.3%)	962 (55.3%)
			dTGA (1 subject)	34 (29.8%)	1258 (29.8%)
	**Validation (5 subjects)**		Healthy (4 subjects)	149 (8.8%)	5513 (8.8%)
			dTGA (1 subject)	40 (35.1%)	1480 (35.1%)
	**Testing (5 subjects)**		Healthy (3 subjects)	117 (7.0%)	4329 (7.0%)
			ToF (1 subject)	21 (44.7%)	777 (44.7%)
			dTGA (1 subject)	40 (35.1%)	1480 (35.1%)

**Table III:** Quantitative Comparison on the 3D Mitea Dataset [17] Showing That Radial View Segmentation Consistently Outperforms Standard Sax, Lax, and 3D Volume-Based Views in Most Metrics. Best Results are Highlighted In Light Green With Bold Font.

Methods and Views		Region-wise HD95 (↓) in 3D		Avg. of ENDO and EPI in 3D		Avg. 2D DSC of ENDO and EPI (slice-by-slice) (↑)
		LV_ENDO_	LV_EPI_	DSC (↑)	MASD (↓)	
**UNet** [8]	3D Volume	6.99 ± 7.41	5.87 ±4.65*	0.875 ± 0.047*	2.30 ± 0.82*	0.756 ± 0.286*
	Short axis (SAX) view	8.95 ± 6.03*	5.56 ±3.11*	0.837 ± 0.062*	2.15 ±0.79	0.738 ± 0.267*
	Long axis (LAX) view	12.68 ± 10.39*	6.51 ±4.65*	0.848 ± 0.057*	2.72 ±1.18*	0.686 ± 0.287*
	**Radial view**	**5.57 ±6.17**	**4.20 ± 4.58**	**0.908 ± 0.034**	**2.08 ± 0.99**	**0.908 ± 0.041**
**SAM** [13]	3D Volume	6.07 ± 3.05*	4.93 ± 2.35*	0.874 ± 0.046*	2.24 ± 0.73*	0.759 ± 0.284*
	Short axis (SAX) view	5.98 ± 4.07*	**2.73 ±1.05**	0.874 ± 0.043*	**1.54 ± 0.49**	0.791 ± 0.230*
	Long axis (LAX) view	7.93 ± 4.44*	3.42 ± 1.78*	0.876 ± 0.039*	2.02 ± 0.62*	0.710 ± 0.270*
	**Radial view**	**4.63 ± 3.44**	3.00 ± 2.47	**0.917 ±0.033**	1.82 ± 0.67	**0.917 ± 0.038**
**MedSAM^†^** [14] (fine-tuned)	Short axis (SAX) view	4.50 ±2.13*	3.44 ± 1.39*	0.878 ± 0.037*	**2.04 ± 0.49**	0.825 ± 0.168*
	Long axis (LAX) view	5.48 ± 2.56*	4.11 ±2.28*	0.878 ± 0.034*	2.50 ± 0.56*	0.795 ±0.184*
	**Radial view**	**4.07 ± 2.42**	**2.98 ± 1.73**	**0.920 ± 0.027**	2.21 ± 0.63	**0.921 ± 0.032**

* Indicates Statistically Significant Difference (*p* < 0.05, Paired T-Test) Compared to the Radial View

**ToF:** Teralogy of Fallot and **dTGA:** Dextro-transposition of the great arteries.

**Table IV:** Quantitative Results on the Feecho4D and Mitea Datasets [17], Comparing the Proposed Data- and Geometry-Driven (Dgd) Scope-Net, Including Ablation Variants, With Purely Data-Driven (Dd) Models. Best Results are Highlighted in Light Green With Bold Font.

Segmentation methods (Purely Data-driven (DD)? or Data+geometry-driven (DGD)?)	Region-wise HD95 (↓)			Average over ENDO and EPI regions		
	LV_MYO_	LV_ENDO_	LV_EPI_	DSC	MASD	PIA
**Our FeEcho4D Dataset**						
UNet-like backbone (DD)	13.1 ± 10.3*	10.1 ±9.33*	9.31 ± 9.36*	0.838 ± 0.140*	6.01 ± 3.20*	1.39 ±4.61*
With prompt only (SAM [13]) (DD)	8.08 ± 3.06*	6.06 ± 2.97*	5.24 ± 2.97*	0.881 ± 0.040*	4.71 ± 1.92*	0.026 ± 0.217*
FCRA only (DGD)	9.44 ± 4.80*	6.69 ± 3.84*	6.43 ± 3.92*	0.874 ± 0.048*	5.07 ±2.08*	0.018 ± 0.206*
ISAI only (DGD)	9.88 ±7.14*	7.52 ± 6.93*	6.82 ± 6.02*	0.872 ± 0.064*	5.18 ±2.37*	0.291 ± 2.41*
I^2^UNet (DD)	12.8 ± 12.7*	7.64 ± 7.10*	9.05 ± 10.9*	0.861 ± 0.095*	5.65 ± 3.26*	0.545 ± 3.37*
UNETR++ (DD)	10.8 ± 8.44*	7.91 ± 7.49*	7.55 ± 7.80*	0.861 ± 0.070*	5.57 ±2.76*	0.291 ± 2.91*
**Our SCOPE-Net** (DGD)	**7.46 ± 3.31**	**5.40 ±3.13**	**4.83 ±3.14**	**0.893 ± 0.044**	**4.29 ±1.71**	**0.004 ± 0.092**
**Public MTTEA Dataset**						
UNet-like backbone (DD)	7.30 ± 3.55*	5.57 ±6.17*	4.20 ± 4.58*	0.908 ± 0.034*	2.08 ± 0.99*	0.749 ± 2.35*
SAM (DD)	6.49 ± 2.20*	4.63 ± 3.44*	3.00 ± 2.47*	0.917 ±0.033*	1.82 ± 0.67	0.050 ± 0.060*
I^2^UNet (DD)	8.03 ± 3.59*	5.54 ± 3.47*	4.08 ± 3.07*	0.910 ± 0.035*	2.47 ±0.85*	0.012 ± 0.075*
UNETR++ (DD)	8.84 ±8.12*	6.93 ±9.81*	5.12 ±8.91*	0.907 ± 0.041*	2.56 ± 1.48*	0.286 ± 1.59*
**Our SCOPE-Net** (DGD)	**5.92 ± 2.37**	**3.96 ± 2.48**	**2.48 ± 1.95**	**0.921 ±0.033**	**1.54 ±0.37**	**5e –4 ± 2e –2**

* Indicates a Statistically Significant Difference (*p* < 0.05, Paired T-Test) Compared to Scope-Net. Fcra: Flip-Consistent Radial Attention; **ISAI:** Inter-Slice Augmentation Invariance

**Table V:** Comparison of 3D Reconstruction and Cardiac Biomarker Estimation Across Input Modalities, Reconstruction Methods, and Segmentation Networks on the Mitea Dataset. Best Results are Highlighted in Light Green With Bold Font

Input	Reconstruction	Segmentation	DSC (↑)	CD (↓)	Correlation (%) (↑)			Infer Time (↓)	Flops (GPU Memory) (↓)
					V_stroke_	EF	GLS		
2D long axis (LAX)	Simpson’s	UNet2D	0.831 ± 0.051	–	63.9	64.4	31.8	10 ms	51*G* (5.4 *GB*)
3D volume	–	UNet3D	0.875 ± 0.047	–	74.8	73.1	10.8	85 ms	79 *G* (9.9 *GB*)
	–	UNETR++	0.915 ± 0.021	–	82.7	81.9	55.6	69 ms	50 *G* (5.4 *GB*)
2.5D Radial	Graph-Harmonic Deformation (GHD)	UNet2D	0.911 ± 0.054	1.21 ±0.70	51.8	57.2	81.8	195 s	SCOPE-Net: 56 *G* (5.7 *GB*) UNet2D: 51*G* (5.4 *GB*) I^2^UNet: 19 *G* (3.4 *GB*) UNETR++: 41*G* (5.1*GB*)
		I^2^UNet	0.926 ± 0.057	1.01 ±0.75	62.9	67.3	76.6		
		UNETR++	0.918 ± 0.060	1.11 ±0.80	62.0	55.1	68.6		
		SCOPE-Net	**0.959 ± 0.026**	**0.47 ± 0.29**	**89.7**	**88.8**	**91.1**		
	Non-rigid Iterative Closest Point (NrlCP)	UNet2D	0.724 ± 0.099	4.57 ± 1.66	14.9	15.9	**6.7**	100 s	
		I^2^UNet	0.801 ± 0.040	2.39 ± 0.48	43.0	42.0	43.7		
		UNETR++	0.710 ±0.111	4.70 ± 1.78	36.8	2.9	48.7		
		SCOPE-Net	0.807 ± 0.080	2.71 ± 1.12	40.8	28.1	53.1		
	Non-rigid Coherent Point Drift (NrCPD)	UNet2D	0.712 ± 0.079	4.78 ± 1.36	10.5	7.3	6.8	30 s	
		I^2^UNet	0.770 ± 0.043	3.02 ± 0.59	37.7	16.6	27.1		
		UNETR++	0.757 ± 0.099	3.15 ± 1.04	41.9	24.8	50.7		
		SCOPE-Net	0.801 ± 0.075	2.90 ±1.13	50.2	46.9	33.2		
	Marching Cube	UNet2D	0.770 ± 0.066	1.27 ±0.31	34.0	65.6	60.8	23 s	
		I^2^UNet	0.835 ± 0.045	1.32 ±0.35	84.3	79.8	60.3		
		UNETR++	0.827 ± 0.044	1.36 ± 0.34	68.6	70.2	36.0		
		SCOPE-Net	0.868 ± 0.056	1.07 ± 0.44	82.3	83.4	58.6		
